# Clinical and Genomic Epidemiology of *mcr-9*-Carrying Carbapenem-Resistant *Enterobacterales* Isolates in Metropolitan Atlanta, 2012 to 2017

**DOI:** 10.1128/spectrum.02522-21

**Published:** 2022-07-20

**Authors:** Ahmed Babiker, Chris Bower, Joseph D. Lutgring, Robert A. Petit, Jessica Howard-Anderson, Uzma Ansari, Gillian McAllister, Michelle Adamczyk, Erin Breaker, Sarah W. Satola, Jesse T. Jacob, Michael H. Woodworth

**Affiliations:** a Division of Infectious Diseases, Department of Medicine, Emory University School of Medicine, Atlanta, Georgia, USA; b Department of Pathology and Laboratory Medicine, Emory University School of Medicine, Atlanta, Georgia, USA; c Georgia Emerging Infections Program, Decatur, Georgia, USA; d Atlanta VA Medical Center, Decatur, Georgia, USA; e Foundation for Atlanta Veterans Education and Research, Decatur, Georgia, USA; f Division of Healthcare Quality Promotion, Centers for Disease Control and Prevention, Atlanta, Georgia, USA; g Theiagen Genomics, Highlands Ranch, Colorado, USA; h Goldbelt C6, LLC, Chesapeake, Virginia, USA; University at Albany, State University of New York

**Keywords:** healthcare epidemiology, next-generation sequencing, CRE, MDR, AMR, antimicrobial resistance, carbapenem-resistant *Enterobacterales*, multidrug resistance

## Abstract

Colistin is a last-resort antibiotic for multidrug-resistant Gram-negative infections. Recently, the ninth allele of the mobile colistin resistance (*mcr*) gene family, designated *mcr-9,* was reported. However, its clinical and public health significance remains unclear. We queried genomes of carbapenem-resistant *Enterobacterales* (CRE) for *mcr-9* from a convenience sample of clinical isolates collected between 2012 and 2017 through the Georgia Emerging Infections Program, a population- and laboratory-based surveillance program. Isolates underwent phenotypic characterization and whole-genome sequencing. Phenotypic characteristics, genomic features, and clinical outcomes of *mcr-9*-positive and -negative CRE cases were then compared. Among 235 sequenced CRE genomes, 13 (6%) were found to harbor *mcr-9*, all of which were Enterobacter cloacae complex. The median MIC and rates of heteroresistance and inducible resistance to colistin were similar between *mcr-9*-positive and -negative isolates. However, rates of resistance were higher among *mcr-9*-positive isolates across most antibiotic classes. All cases had significant health care exposures. The 90-day mortality was similarly high in both *mcr-9*-positive (31%) and -negative (7%) CRE cases. Nucleotide identity and phylogenetic analysis did not reveal geotemporal clustering. *mcr-9*-positive isolates had a significantly higher number of median [range] antimicrobial resistance (AMR) genes (16 [4 to 22] versus 6 [2 to 15]; *P* < 0.001) than did *mcr-9*-negative isolates. Pangenome tests confirmed a significant association of *mcr-9* detection with mobile genetic element and heavy metal resistance genes. Overall, the presence of *mcr-9* was not associated with significant changes in colistin resistance or clinical outcomes, but continued genomic surveillance to monitor for emergence of AMR genes is warranted.

**IMPORTANCE** Colistin is a last-resort antibiotic for multidrug-resistant Gram-negative infections. A recently described allele of the mobile colistin resistance (*mcr*) gene family, designated *mcr-9,* has been widely reported among *Enterobacterales* species. However, its clinical and public health significance remains unclear. We compared characteristics and outcomes of *mcr-9*-positive and -negative CRE cases. All cases were acquired in the health care setting and associated with a high rate of mortality. The presence of *mcr-9* was not associated with significant changes in colistin resistance, heteroresistance, or inducible resistance but was associated with resistance to other antimicrobials and antimicrobial resistance (AMR), virulence, and heavy metal resistance (HMR) genes. Overall, the presence of *mcr-9* was not associated with significant phenotypic changes or clinical outcomes. However, given the increase in AMR and HMR gene content and potential clinical impact, continued genomic surveillance of multidrug-resistant organisms to monitor for emergence of AMR genes is warranted.

## INTRODUCTION

With the rise of carbapenem-resistant organisms over the past few decades (1, 2), polymyxins (colistin or polymyxin B) remain last-resort antibiotics for multidrug-resistant (MDR) Gram-negative infections (3). While concerns regarding their efficacy and nephrotoxicity (4) have relegated polymyxins to the second or third line of the antibiotic armamentarium (5), these agents remain listed as critically important antibiotics by the WHO and are widely used globally.

In 2015, a colistin resistance gene localized on a plasmid was designated mobilized colistin resistance 1 (*mcr-1*) (6). The *mcr-1* gene encodes a transferase that adds a phosphoethanolamine residue to cell membrane lipid A, altering the binding site of colistin and consequently leading to colistin resistance (6). Since its initial description in Escherichia coli (6), multiple *mcr* alleles (*mcr*-*2* to *mcr*-*10.1*) have been described (7). The *mcr-9* allele was reported in 2019 and is most similar to *mcr-3* among previously described *mcr* alleles (8). A recent search of publicly available sequence databases revealed a wide global distribution of *mcr-9*-harboring isolates, across six continents and in at least 9 *Enterobacterales* species (7, 9). However, it is most commonly detected among Enterobacter species (9).

In the United States, the initial wave of carbapenem-resistant *Enterobacterales* (CRE) was predominantly driven by proliferation of KPC-harboring Klebsiella pneumoniae (1, 10); however, as demonstrated in recent reports, a second wave of CRE in the United States seems to be driven by the rise of Enterobacter species (1, 11). Genomic analysis indicates this second wave is associated with a high degree of clonal diversity among isolates (12). With the increasing spread of carbapenem-resistant Enterobacter, the dissemination of *mcr-9* is highly probable. Despite its global distribution, the impact of *mcr-9* on colistin phenotypic susceptibility remain unclear. Moreover, its association with patient clinical outcomes or potential for outbreaks of public health concern is yet to be examined.

The Centers for Disease Control and Prevention (CDC)-funded Georgia Emerging Infections Program (GA EIP) performs active, population- and laboratory-based surveillance for CRE isolated from sterile sites or urine in metropolitan Atlanta, GA (population ~4 million). We aimed to estimate the frequency of *mcr-9* among CRE cases within the GA EIP catchment area and to compare clinical outcomes and microbiological, genomic, and clinical characteristics of *mcr-9*-positive and *mcr-9*-negative cases.

## RESULTS

### The *mcr-9* allele was infrequently detected among GA EIP isolates between 2012 and 2017.

**FIG 1:**
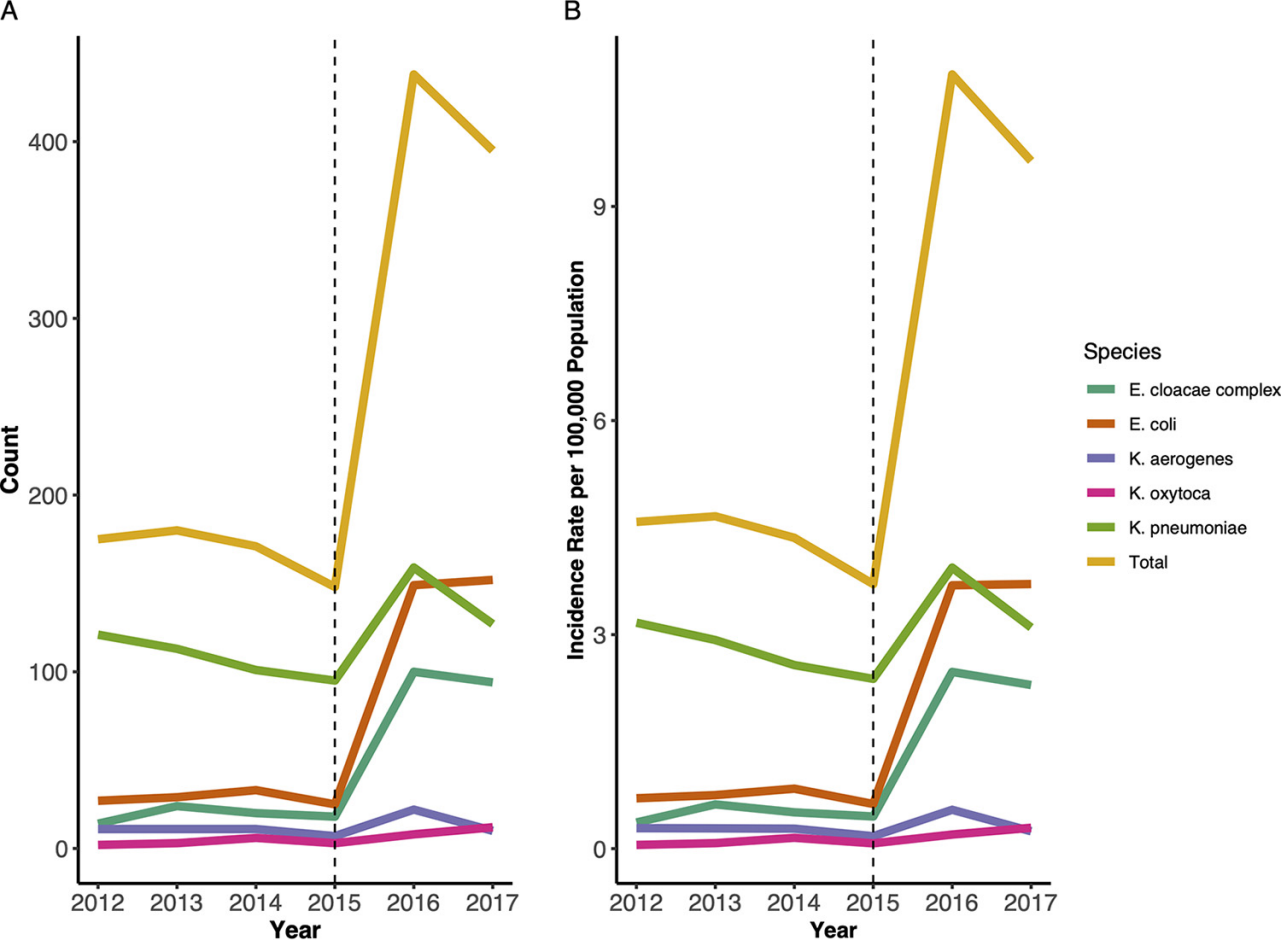
Carbapenem-resistant *Enterobacterales* (CRE) count (A) and crude annual incidence per 100,000 population (B) by species across the Georgia Emerging Infections Program from 2012 to 2017. Beginning in 2016, the phenotypic CRE case definition was changed to resistance to ≥1 carbapenem (now including ertapenem) with no cephalosporin parameter.

Between 2012 and 2017, the GA EIP identified 1,507 incident CRE cases; 716 (47.5%) were K. pneumoniae, 415 (27.5%) were Escherichia coli, 270 (17.9%) were Enterobacter cloacae complex, 72 (4.8%) were Klebsiella aerogenes (formerly Enterobacter), and 34 (2.3%) were Klebsiella oxytoca. The overall crude annual CRE incidence across GA EIP increased from 4.6 to 9.6 per 100,000 population from 2012 to 2017. Carbapenem-resistant E. cloacae complex incidence, in particular, increased from 0.37 to 2.3 per 100,000 population during the study period. This increase coincided with revision of the CDC case surveillance definition for CRE (13) (Fig. 1A and B and see also Table S1 and Fig. S1 in the supplemental material).

A convenience sample of 384 isolates which met the GA EIP CRE case definition was sent to the CDC for further characterization. Of the 384, 235 (61%) underwent whole-genome sequencing (WGS). Among 235 sequenced CRE isolates, 13 (6%) were found to harbor *mcr-9*, all of which were E. cloacae complex. All remaining sequenced *mcr-9*-negative E. cloacae complex isolates (*n* = 14) were included as a comparative group, yielding a total number of 27 E. cloacae complex isolates.

### Microbiology characteristics.

Following collection, isolates underwent reference antimicrobial susceptibility testing by broth microdilution (BMD) at the CDC. Of the E. cloacae complex isolates that underwent WGS from 2012 to 2017, 22 isolates (81.7%, 22/27) were confirmed to be carbapenem resistant. Carbapenem resistance rates were similar between *mcr-9*-positive and -negative cases (84.6% [11/13] versus 78.6% [11/14]; *P* = 1.00). Fluoroquinolone resistance was significantly higher among *mcr-9*-positive isolates than *mcr-9*-negative isolates (100% [13/13] versus 57.1% [8/14]; *P* = 0.03). This contributed to a higher proportion of isolates being classified as difficult-to-treat resistance (DTR) (14). Overall, 48.1% (13/27) were classified as harboring DTR (14, 15). with *mcr-9* isolates having higher-rates of DTR (61.5% [8/13] versus 35.7% [5/14]; *P* = 0.34). Similarly, rates of aminoglycoside, tetracycline, and trimethoprim-sulfamethoxazole resistance were higher among *mcr-9*-positive isolates than *mcr-9*-negative isolates (Table S2).

The median (range of concentrations tested) colistin MIC for all E. cloacae complex isolates was 0.5 μg/mL (≤0.25 to >8.0). Proportions of resistance, heteroresistance, and inducible resistance were 11.1% (3/27), 48.1% (13/27), and 14.8% (4/27), respectively. There was no significant difference in colistin MIC, heteroresistance, or inducible resistance between *mcr-9*-positive and -negative isolates (Table 1). Of the three E. cloacae complex isolates which were colistin resistant by BMD, none were *mcr-9* positive, although one was positive for *mcr-10.1*.

**TABLE 1:** Carbapenem-resistant E. cloacae complex clinical and microbiological characteristics

Characteristic	No. (%) for category:			*P* value
	All (*n* = 27)	*mcr-9* positive (*n* = 13)	*mcr-9* negative^*a*^ (*n* = 14)	
Culture source				0.33
Urine	24 (88.9)	12 (92.3)	12 (85.7)	
Blood	2 (7.4)	1 (7.7)	1 (7.1)	
Peritoneal fluid	1 (3.7)	0 (0.0)	1 (7.1)	
Yr				0.62
2012	1 (3.7)	1 (7.7)	0 (0.0)	
2013	8 (29.6)	2 (15.4)	6 (42.9)	
2014	2 (7.4)	1 (7.7)	1 (7.1)	
2015	3 (11.1)	3 (23.1)	0 (0.0)	
2016	9 (33.3)	4 (30.8)	5 (35.7)	
2017	4 (14.9)	2 (15.4)	2 (14.3)	
Infection onset				0.71
Hospital onset	3 (11.1)	2 (15.4)	1 (7.1)	
Healthcare-associated community onset	10 (37.0)	4 (30.8)	6 (42.9)	
Long-term care facility onset	14 (51.9)	7 (53.8)	7 (50.0)	
Microbiology characteristic				
Colistin MIC (median [range])^*b*^	0.5 [<0.25–>0.8]	0.5 [<0.25–1.00]	0.5 [<0.25–>8.0]	0.11
Resistant	3 (11.1)	0	3 (21.4)	0.25
Heteroresistant	13 (48.1)	5 (38.5)	8 (57.1)	0.56
Inducible resistance	4 (14.8)	2 (15.4)	2 (14.3)	1.00
Difficult-to-treat resistance	13 (48.1)	8 (61.5)	5 (35.7)	0.34
Outcomes				
Hospitalization within 29 days after culture	10 (37.0)	5 (38.5)	5 (35.7)	0.86
ICU admission^*c*^^,^^*d*^	4 (40.0)	2 (40.0)	2 (40.0)	1.00
In-hospital mortality^*d*^	1 (10.0)	1 (20.0)	0 (0.0)	0.59
90-day mortality	5 (18.5)	4 (30.8)	1 (7.1)	0.28

a One *mcr-10*-positive isolate.

b MIC units are micrograms per milliliter.

c Any ICU admission 7 days before or 6 days after specimen collection.

d Among 10 hospitalized patients, 5 in each group (*mcr-9* positive and negative).

### Clinical characteristics of *mcr-9*-positive and -negative CRE cases.

E. cloacae complex isolates were commonly isolated from urine (88.9%, 24/27), followed by blood (7.4%, 2/27) and peritoneal fluid (3.7%, 1/27). All cases had significant health care exposures, with 14 cases (51.9%) of long-term care facility onset, 10 (37.0%) of health care-associated community onset, and 3 (11.1%) of hospital onset. Ten patients (37.0%) were hospitalized at time of culture or within 29 days of CRE culture. Among the 10 hospitalized patients, 3 (30.0%) patients were admitted to the intensive care unit (ICU) within 7 days of culture and one patient died during the period of hospitalization (10.0%). Among hospitalized patients with available follow-up data (*n* = 7), 42.8% (3/7) were readmitted within 30 days. Overall unadjusted all-cause 90-day mortality was 18.5% (5/27). No clinical characteristics and outcomes were significantly different, and most were numerically similar among *mcr-9*-positive and -negative cases. Ninety-day mortality was higher among *mcr-9*-positive cases (30.7% [4/13] versus 7.1% [1/14]) than *mcr-9*-negative cases, but this was not statistically significant (*P* = 0.28).

### Genomic analysis.

To expand the analytic genome set, a comparator cohort of nine publicly available clinical and environmental carbapenem-resistant E. cloacae complex (three *mcr-9* positive, six *mcr-9* negative) genomes were downloaded from the National Center for Biotechnology Information (NCBI) and included in further comparative genomic analyses. Review of associated metadata revealed the isolates to have been collected between January 2012 and December 2016 at the National Institutes of Health (NIH) Clinical Center (Bethesda, MD) (PRJNA430442) (16). In addition, 582 publicly available global E. cloacae complex genomes were included in the phylogenetic analysis.

**FIG 2:**
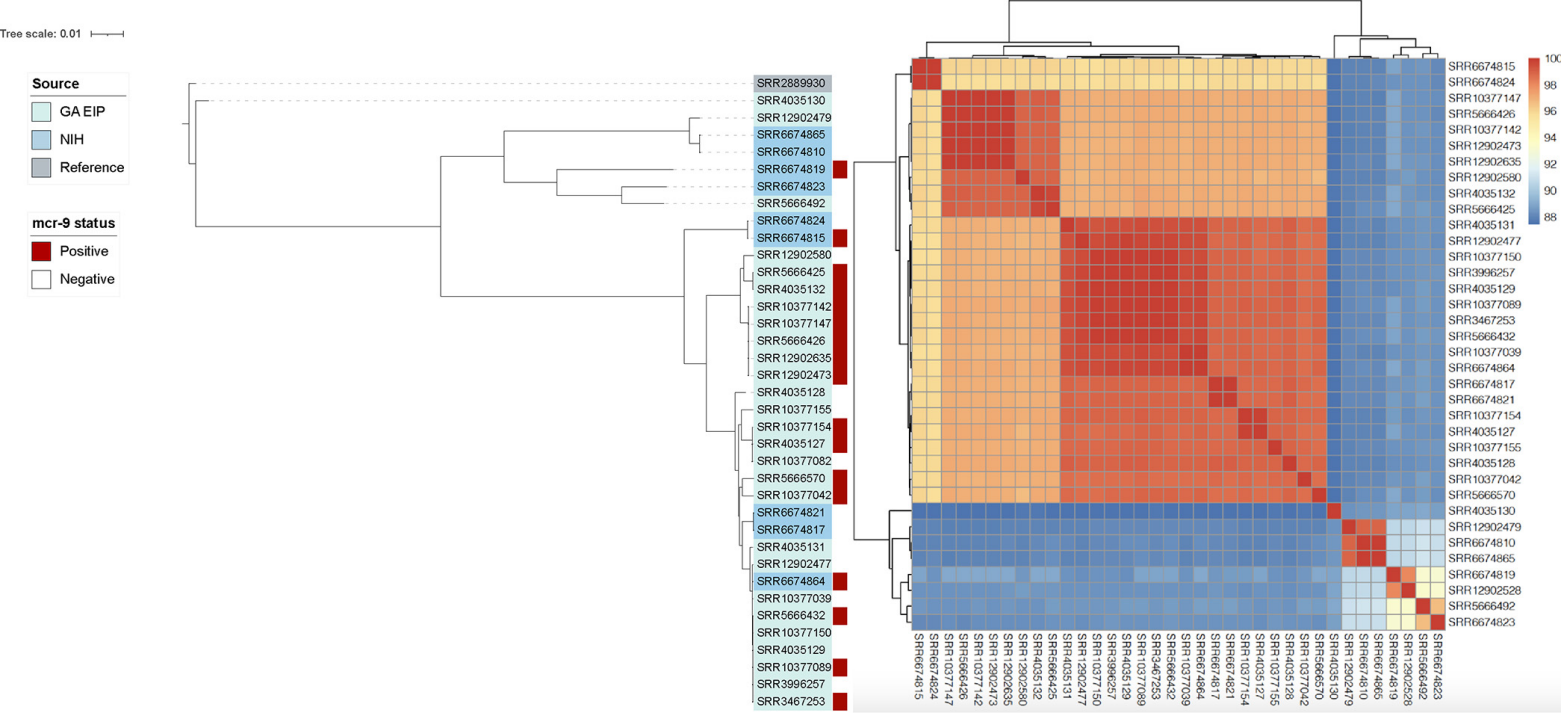
Phylogeny (left) and average nucleotide identity heatmap (right) of *mcr-9*-positive (*n* = 13) and *mcr-9*-negative (*n* = 14) E. cloacae complex genomes from the Georgia Emerging Infections Program in addition to 9 available E. cloacae complex genomes (three *mcr-9* positive, six *mcr-9* negative) from the National Institutes of Health. A phylogenetic tree based on a core gene alignment containing 1,904 genes defined using Roary v3.13.0 was generated using IQtree v2.0.3. A maximum likelihood tree was generated by running 1,000 bootstrap replicates under the generalized time-reversible model of evolution. The tree was visualized and annotated using Interactive Tree of Life (iTOL) v4. Pairwise comparisons of average nucleotide identity on the assembled genomes were performed with the Mashmap method using fastANI v1.32. GA EIP, Georgia Emerging Infections Program; NIH, National Institutes of Health.

The 13 *mcr-9*-positive E. cloacae complex isolates were obtained from 10 distinct GA EIP facilities across the study period (2012 to 2017). Average nucleotide identity pairwise comparisons revealed three distinct clusters (Fig. 2). The clustering did not appear to be related to geographical location or year of isolation. NIH isolates clustered with GA EIP isolates, and GA EIP isolates did not cluster by facility or year (Fig. S2). Their distribution throughout the genome phylogeny suggested that the *mcr-9*-positive isolates were largely genetically distinct from one another. Among the total set of 609 genomes, 16% (98/609) were *mcr-9* positive. Both the local GA EIP and global *mcr-9*-positive genomes were distributed throughout the genome phylogeny. A notable exception were genomes DRX055644 to DRX055660, which were sequenced as part of an ongoing outbreak at a burn center (17); many of the genomes had a pairwise single nucleotide polymorphism (SNP) distance of <50 SNPs (Fig. S3 and Table S4).

**FIG 3:**
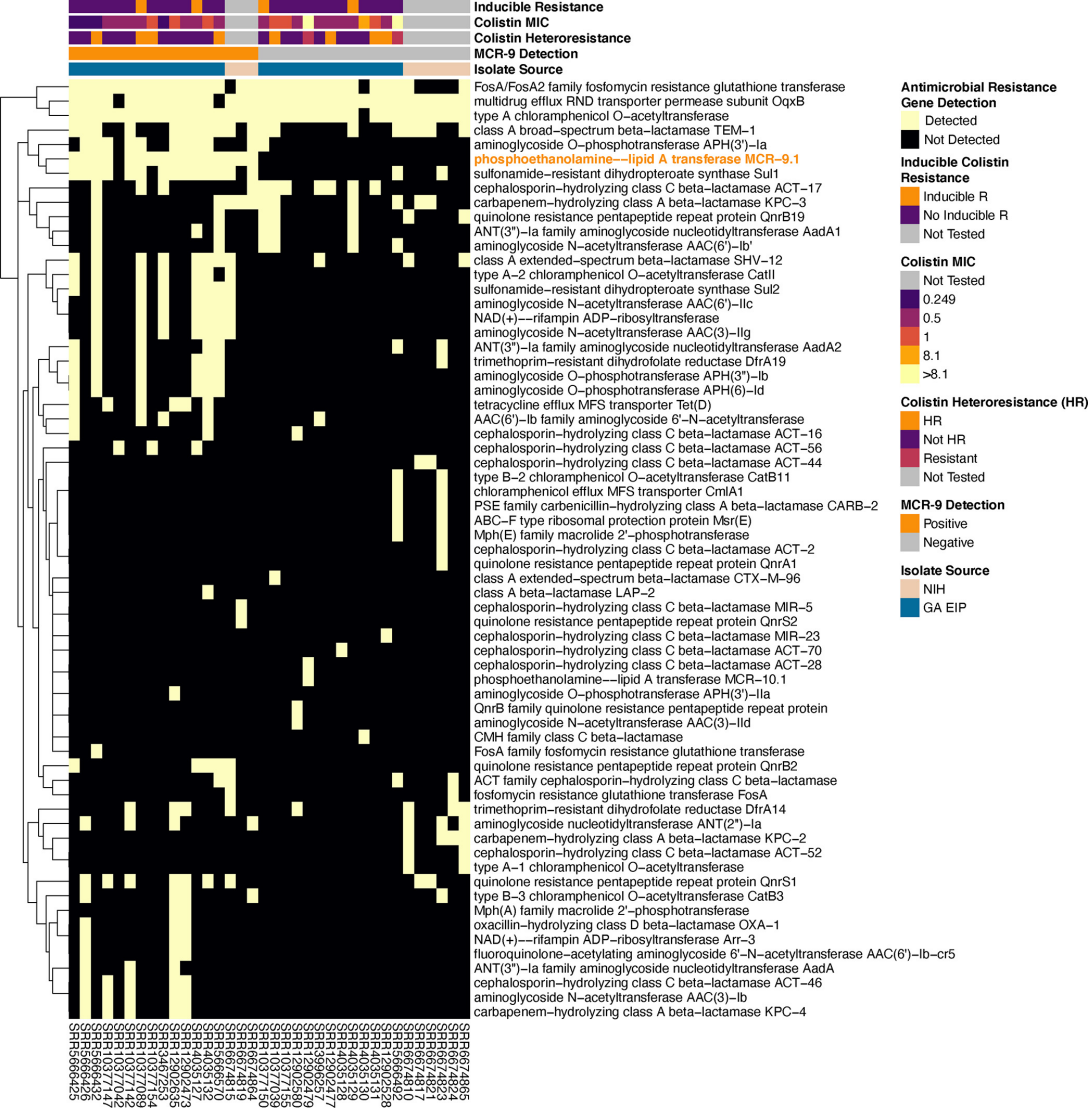
Antimicrobial resistance gene heatmap of *mcr-9*-positive (*n* = 13) and *mcr-9*-negative (*n* = 14) E. cloacae complex genomes from the Georgia Emerging Infections Program in addition to 9 available E. cloacae complex genomes (three *mcr-9* positive, six *mcr-9* negative) from the National Institutes of Health. Genomes were annotated using Prodigal v2.6.3, and antimicrobial resistance gene content was assessed using AMRFinder. Antimicrobial resistance gene presence/absence heatmaps were created using the package pheatmap on R version 4.0.2 (Vienna, Austria) and the RStudio interface version 1.3.1073 (Boston, MA, USA).

Median [range] antimicrobial resistance (AMR) gene content (excluding *mcr-9*) was significantly higher among *mcr-9*-positive isolates than *mcr-9*-negative isolates (16 [4 to 22] versus 6 [2 to 15]; *P* < 0.001) (Fig. 3). Among the three isolates with elevated colistin MICs, no point mutations in *pmrA/pmrB*, a two-component system regulator of lipopolysaccharide (the target site of colistin) modifications, were detected. Pangenome-wide association tests revealed a significant association of *mcr-9* detection with the detection of mobile genetic element (MGE)-associated genes such as *repB*, *parM*, and *hns2*; heavy metal resistance (HMR) genes such as *arsC2*, *arsB2*, *fieF2*, *pcoE2*, and *merA*; and virulence genes such as *hipA* (Table 2, Table S2, and Fig. S3). Taken together, these comparative genomic analyses across two sites with *mcr-9*-positive E. cloacae complex isolate draft genomes confirmed the colocalization of *mcr-9* with plasmid-mobilized heavy metal resistance genes but did not provide evidence of a high-identity outbreak cluster in space or time.

**TABLE 2:** Highest-ranking genes for association with *mcr-9* presence

Gene^*a*^	Comment	Odds ratio	Bonferroni-adjusted *P* value
*smc*	Chromosome partition protein Smc	∞	1.60E−06
*dcm2*	DNA-cytosine methyltransferase	∞	1.60E−06
*pcoE2*	Putative copper-binding protein PcoE	∞	2.88E−05
*hns2*	DNA-binding protein H-NS, plasmid	∞	3.36E−05
group_10390	Tn*3* family transposase ISEc63	∞	3.36E−05
*hipA*	Serine/threonine-protein kinase toxin HipA	∞	3.36E−05
*rcnR2*	Transcriptional repressor RcnR	∞	0.00030219
*hha2*	Hemolysin expression-modulating protein Hha	∞	0.00036934
*uvrD2*	DNA helicase II	∞	0.00036934
*parM*	Plasmid segregation protein	∞	0.00036934
*higB-1*	Toxin HigB-1	∞	0.00036934
group_1846	Tn*3* family transposase ISEc63	∞	0.00036934
*dam2*	DNA adenine methylase	∞	0.00036934
*repB*	RepFIB replication protein A	∞	0.00036934
*traC*	Protein TraC	∞	0.00036934
group_7173	Stable plasmid inheritance protein	∞	0.00036934
*yjcD*	Putative ATP-dependent DNA helicase YjcD	304	0.00054522
*umuD2*	Protein UmuD	∞	0.00283164
*dsbC_2*	Thiol:disulfide interchange protein DsbC	∞	0.00283164
*virB*	Virulence regulon transcriptional activator VirB	∞	0.00283164
*umuC_3*	Protein UmuC	∞	0.00283164
group_7174	IS*110* family transposase ISEsa2	142.5	0.00540586
*merA*	Mercuric reductase	∞	0.01698985
*fieF_2*	Ferrous-iron efflux pump FieF	90.7	0.03283809
*arsB_2*	Arsenical pump membrane protein	67.5	0.04368339
group_7063	ISNCY family transposase ISEsa1	67.5	0.04368339
group_8953	ISNCY family transposase ISBcen27	67.5	0.04368339
*arsH*	NADPH-dependent FMN^*b*^ reductase ArsH	67.5	0.04368339
*arsC2*	Arsenate reductase	67.5	0.04368339

a Hypothetical proteins not included.

b FMN, flavin mononucleotide.

## DISCUSSION

Among 235 CRE isolates collected through a comprehensive, population-based surveillance program targeting the most common CRE species, we found a low prevalence of *mcr-9*, all of which was detected in Enterobacter cloacae isolates. Our phylogenetic analyses revealed a genetically diverse *mcr-9*-positive CRE population, suggesting sporadic carriage rather than clonal spread. Using multimodal phenotypic testing, we were unable to detect impacts of *mcr-9* on colistin susceptibility; however, genomic analysis revealed an association with increased AMR, HMR, and virulence genes. In addition, our *mcr-9*-containing CRE isolates were exclusively acquired in health care settings, with a trend toward increased mortality. Since their initial description, recognition of *mcr* genes associated with colistin resistance has spread rapidly across the globe (7). Our study of *mcr-9*-harboring CRE cases provides unique insights into the phenotypic and genomic implications of *mcr-9* and is one of the first to examine clinical outcomes.

Whether *mcr-9* confers colistin resistance has been debated (18). The first isolate identified to harbor *mcr-9* was also susceptible to colistin, but the allele was found to confer resistance to colistin when cloned into a colistin-susceptible E. coli strain and expressed under the control of an isopropyl-β-d-thiogalactopyranoside (IPTG)-induced promoter. However, this was only at 1, 2, and 2.5 mg/L, not at 5 mg/L, of colistin (8). Kieffer et al. later reported that *mcr-9* expression was inducible in the presence of colistin when located upstream of the two-component sensor kinase system *qseBC* (18). This two-component signaling network allows bacteria to sense and respond to their changing environments. In particular, the *qseC* and *qseB* genes encode a histidine kinase sensor (*qseC*) and its cognate partner (*qseB*). The *qseBC* system has been shown to interact with *pmrA/pmrB*, to induce resistance to colistin (19). However, a study of *mcr-9*-containing isolates from retail meat conducted by the National Antimicrobial Resistance Monitoring System (NARMS) found all 105 isolates (99 Salmonella enterica and 6 E. coli) tested to be susceptible to colistin, including 10 isolates with *qseBC* (20), indicating that the previously demonstrated impact of *qseBC* on *mcr-9* expression and colistin resistance may be dependent on strain backgrounds, as originally demonstrated in E. coli (18). Among clinical CRE isolates, we found the presence of *mcr-9* was not associated with frank or inducible colistin resistance. Furthermore, our study is the first to examine the association of *mcr-9* with heteroresistance. Heteroresistance is a largely unrecognized form of antibiotic resistance where only a subset of cells within a bacterial population are resistant to a given drug (21). These resistant cells can be selected for in the presence of the antibiotic and cause colistin treatment failures *in vivo* (22). In a multisite surveillance study of colistin heteroresistance among CRE, Enterobacter spp. and, in particular, E. cloacae displayed the highest proportion of colistin heteroresistance (23). However, here we found no association between *mcr-9* and colistin heteroresistance.

Carbapenem-resistant Gram-negative bacteria are a public health threat broadly prioritized by public health organizations (24). Given the limited therapeutic options, morbidity and mortality rates are increased disproportionately compared to infections caused by susceptible bacteria. We observed high 90-day mortality rates, but these were similar to reported CRE mortality rates at other U.S. academic centers (1). However, there was a nonsignificant numerically higher rate of mortality associated with *mcr-9*-positive isolates. This association should be further evaluated in larger studies, with adjustment for potentially confounding variables associated with mortality such as severity of illness, age, and comorbidities as our study’s small size may underestimate differences in mortality (1). This finding may be related to the increase in phenotypic resistance and AMR gene content associated with *mcr-9*. A similar finding was previously reported describing 1,035 *mcr-9*-containing isolates in which 97% (1003/1035) were classified as multidrug resistant (MDR) (7). This increased AMR gene content renders isolates not only carbapenem resistant but also with DTR, further limiting therapeutic options (14). DTR is a clinically relevant and functional classification of resistance which signifies *in vitro* resistance to all high-efficacy, low-toxicity (or first-line) agents and is associated with worse clinical outcomes than those of carbapenem-resistant phenotypes (14). Moreover, genome-wide association studies revealed a difference in key virulence genes such as *hipA*, a eukaryote-like serine threonine kinase that inhibits cell growth and induces bacterial persistence (25).

We found the presence of *mcr-9* to be associated with HMR genes such as *arsA* and *merA*, conferring arsenic and mercury resistance, respectively. There has been increasing evidence for the coselection of AMR and HMR genes through either coresistance or cross-resistance (26). Coresistance occurs when AMR and HMR genes are carried on the same mobile genetic element. IncH12 plasmids, which frequently harbor HMR genes (8, 27), have been found to be the predominant replicon type carrying *mcr-9* and frequently demonstrated in our isolates. Hospital wastewater is an increasingly recognized reservoir for resistant Gram-negative organisms that cause health care-associated infections (28), and HMR genes may allow for continued persistence in the environment (29). While the community setting is starting to represent an increased source of multidrug-resistant infections (30), the health care setting still represents a major risk for MDR acquisition, as was seen among our cohort.

Our study combines detailed epidemiological, clinical, phenotypic, and genomic data to examine the significance of *mcr-9* but has some limitations. First, we could not do a full interrogation of *mcr-9*-containing plasmids, due to limitations of short-read sequencing. However, prior studies have significantly characterized the genomic background of *mcr-9*-containing plasmids (9). Second, our study did not include Salmonella species, which are a major reservoir for *mcr-9*, or other *Enterobacterales* species such as *Citrobacter* (7), and our findings may not be generalizable to these species. However, our data set of 235 includes the most common and significant clinical CRE species (1, 24) and is one of the few studies carried out on clinical human isolates (20). Third, while we assessed for the presence of the *pmrA/pmrB* regulatory system (31), we did not include the assessment of the two-component system *qsceBC* which has been shown to influence *mcr-9* expression and colistin MIC results. Fourth, while our overall cohort is from a population-based surveillance program, the collected and sequenced isolates represent a convenience sample, which may limit generalizability, and our sample size was not powered to control for important variables such as source of infection, severity of illness, and treatment received and to detect clinical outcomes.

In conclusion, *mcr-9* may not have actionable public health implications as do other *mcr* alleles, most of which consistently display colistin resistance. However, given the increased AMR and HMR gene content, continued genomic surveillance of multidrug-resistant organisms to monitor for the emergence of AMR genes such as *mcr-9* is prudent, especially as changes in the up- or downstream genetic context or the accumulation of mutations may impact its ability to confer colistin resistance.

## MATERIALS AND METHODS

CRE cases were identified by routine queries on automated testing instruments in the clinical labs that serve residents of the GA EIP catchment area. Clinical characteristics were obtained through medical record review, all-cause mortality data were obtained through the Georgia Vital Statistics records, and hospital readmission data were obtained through the Department of Public Health’s hospital discharge data sets. Georgia EIP surveillance activities are reviewed and approved by the Emory University Institutional Review Board (IRB00089004).

From 2012 to 2015, a CRE case was defined as an isolate of E. coli, E. cloacae complex, *K*. (formerly Enterobacter) *aerogenes*, K. pneumoniae, or K. oxytoca collected from a normally sterile body site (e.g., bloodstream) or urine that tested nonsusceptible to ≥1 carbapenem (imipenem, meropenem, or doripenem) and resistant to all third-generation cephalosporins tested (ceftriaxone, ceftazidime, and cefotaxime) by testing performed at the local collection microbiology laboratory. Beginning in 2016, the phenotypic case definition was changed to resistance to ≥1 carbapenem (now including ertapenem) with no cephalosporin parameter. Antibiotic susceptibility interpretations were determined using the current Clinical and Laboratory Standards Institute breakpoints (32). Fluoroquinolone resistance was defined as nonsusceptibility (intermediate or resistant) to ≥1 fluoroquinolone. DTR was defined as intermediate or resistant to all reported agents in carbapenem, β-lactam, and fluoroquinolone categories (14, 15).

An incident CRE case was defined as the first CRE isolate from a patient during a 30-day period that met the surveillance definition. All incident CRE cases underwent medical record review using a standardized abstraction form. Both inpatient and outpatient medical records were reviewed for patient demographics, underlying clinical comorbidities, location of culture collection, specimen source, associated infectious syndromes, relevant health care exposures, and patient outcomes. Ninety-day mortality was determined based on matching to vital records.

A convenience sample of CRE isolates is collected annually and submitted to the CDC for further characterization. Isolates that are collected and matched to an incident case with a completed case report form are eligible for shipment. All isolates undergo repeat reference BMD at the CDC followed by whole-genome sequencing using an Illumina MiSeq benchtop sequencer.

All *mcr-9*-positive and a comparative control group of *mcr-9*-negative E. cloacae complex isolates underwent additional population analysis profiling and inducible resistance testing at the Emory Investigational Clinical Microbiology Core as previously described (21). Briefly, all isolates were tested via the population analysis profile (PAP) method. This consists of plating overnight cultures of each isolate onto solid cation-adjusted Mueller-Hinton (MH) agar with or without colistin concentrations of 0.5, 1, 2, 4, 16, 32, and 100 μg/mL. Surviving colonies were enumerated and used to detect colistin-resistant subpopulations characteristic of heteroresistance (21). Inducible resistance testing was performed as previously described (33). Briefly, a single colony of each clinical isolate was grown in cation-adjusted MH broth overnight at 37°C, and cultures were diluted 1:100 in MH broth containing serially increasing concentrations of colistin, starting at the one-half MIC value of the respective isolate and doubling every 24 h until bacterial growth was completely inhibited (with no bacterial growth after spreading 100 μL of the culture on MH agar plates supplemented with the corresponding concentration of colistin). The concentration of colistin at which bacterial growth was completely inhibited was recorded as the final colistin concentration.

### Bioinformatic methods.

Fastq files of Enterobacter cloacae complex isolates of interest were downloaded from the Sequence Read Archive (SRA) repository maintained by the National Center for Biotechnology Information (NCBI) using the fasterq-dump tool from the SRA Toolkit v2.5.7 (https://hpc.nih.gov/apps/sratoolkit.html). Illumina reads were quality filtered using Trimmomatic (34) and assembled *de novo* using SPAdes v3.13 (35). Pairwise comparisons of average nucleotide identity on the assembled genomes were performed with the Mashmap method using fastANI v1.32 (36). Gene sequences were predicted with Prodigal v2.6.3 (37) and annotated with Prokka v1.14.6 (38). Antimicrobial resistance and virulence gene content was assessed using AMRFinder Plus (39). The presence of plasmids and point mutations in housekeeping genes associated with colistin resistance was assessed using the ResFinder and PlasmidFinder web interface with default settings and the E. coli database (40, 41). AMR gene presence/absence heatmaps were created using the package pheatmap on R version 4.0.2 (Vienna, Austria) and the RStudio interface version 1.3.1073 (Boston, MA, USA). Pangenome-wide comparison of core genomes of *mcr-9*-positive to *mcr-9*-negative genomes was completed using Scoary (42). Additional publicly available global E. cloacae complex genomes were downloaded, assembled, and analyzed using the Bactopia pipeline (43). Core genes were defined using PIRATE (44). A phylogenetic tree based on a core gene alignment was generated using IQtree v2.0.3 (45). A maximum likelihood tree was generated by running 1,000 bootstrap replicates under the generalized time-reversible model of evolution. The tree was visualized and annotated using Interactive Tree of Life (iTOL) v4 (46). The core genome pairwise SNP distance for each sample is also calculated with snp-dists (47).

### Statistical analysis.

Annual incidence rates for CRE cases were calculated using the annual U.S. census estimates of the surveillance area population as the denominator. Descriptive analyses were performed to summarize specimen information, health care exposures, outcomes, and microbiological results of incident cases; χ^2^ and Wilcoxon rank sum tests were used to compare groups when applicable. Gene differences were assessed by a *P* value adjusted with Bonferroni’s method for multiple-comparison correction. Statistical analysis was performed using R version 4.0.2 (Vienna, Austria) and the RStudio interface version 1.3.1073 (Boston, MA, USA). A two-sided *P* value of <0.05 was considered statistically significant.

### Data availability.

All local GA sequence data are available on NCBI under BioProject PRJNA288601 (GA isolates). Accession numbers for global accessions are found in Table S5 in the supplemental material.
