# Supplementary material for: Clinical and Genomic Epidemiology of mcr-9-Carrying Carbapenem-Resistant Enterobacterales Isolates in Metropolitan Atlanta, 2012 to 2017
Source: Microbiol Spectr. 2022 Jul 20;10(4):e02522-21. doi: 10.1128/spectrum.02522-21 (PMC9431279; doi:10.1128/spectrum.02522-21)
Supplement: Supplemental file 1 — Supplemental material. Download spectrum.02522-21-s0001.pdf, PDF file, 1.4 MB [file spectrum.02522-21-s0001.pdf]

Supplementary Material

Supplemental Table 1: Definitions of carbapenem-resistant Enterobacterales (CRE) and difficult-to-treat resistance (DTR) used during the study period.

Definition	Period	Species	Susceptibility Phenotype
Initial GA EIP surveillance definition	2011–2015	*Escherichia coli*<br>*Klebsiella pneumoniae*<br>*Klebsiella oxytoca*<br>*Enterobacter cloacae* complex<br>*Klebsiella aerogenes*[a]	Intermediate or resistant to:<br>- Imipenem (MIC ≥2 µg/mL), or<br>- Meropenem (MIC ≥2 µg/mL), or<br>- Doripenem (MIC ≥2 µg/mL)<br><br>AND resistant to[b]:<br>- Ceftazidime (MIC ≥16 µg/mL), and<br>- Ceftriaxone (MIC ≥4 µg/mL), and<br>- Cefotaxime (MIC ≥4 µg/mL)
Revised GA EIP surveillance definition	2016–present	*Escherichia coli*<br>*Klebsiella pneumoniae*<br>*Klebsiella oxytoca*<br>*Enterobacter cloacae* complex<br>*Klebsiella aerogenes*[a]	Resistant to:<br>- Imipenem (MIC ≥4 µg/mL), or<br>- Meropenem (MIC ≥4 µg/mL), or<br>- Doripenem (MIC ≥4 µg/mL), or<br><br>- Ertapenem (MIC ≥2 µg/mL)

DTR	2011–2017	*Enterobacter cloacae* complex	Intermediate or resistant to all tested and reported agents in carbapenem, β-lactam, and fluoroquinolone categories

a. Formerly *Enterobacter aerogenes*

b. If the antibiotics were tested

Abbreviations: GA EIP: Georgia Emerging Infections Program; DTR: difficult-to-treat resistance; MIC: minimum inhibitory concentrations

Supplementary Table 2: Antimicrobial Susceptibility testing of Carbapenem-resistant *E. cloacae* complex by *mcr-9* status

Antibiotic	Median (range) MIC*		Susceptible N (%)		Intermediate N (%)**		Resistant N (%)		*P* value ***
	mcr-9 positive (n=13)	*mcr-9* negative (n=14)	*mcr-9* positive (n=13)	*mcr-9* negative (n=14)	*mcr-9* positive (n=13)	*mcr-9* negative (n=14)	*mcr-9* positive (n=13)	*mcr-9* negative (n=14)	
**Aminoglycosides**									
Amikacin	≤1 (≤1->64)	≤1 (≤1-16)	12 (92.3)	14 (100)	0 (0)	0 (0)	1 (7.7)	0 (0)	0.97
Gentamicin	8 (≤0.25->16)	0.37 (≤0.25->16)	4 (30.8)	11 (78.6)	3 (23.1)	1 (7.1)	6 (46.2)	2 (14.3)	0.04
Tobramycin	8 (≤0.5->16)	≤0.5 (≤0.5 ->16)	3 (23.1)	9 (64.3)	4 (30.8)	2 (14.3)	6 (46.2)	3 (21.4)	0.10
**B-lactams**									
Aztreonam	>64 (64->64)	>64 (32->64)	0 (0)	0 (0)	0 (0)	0 (0)	13 (100)	14 (100)	n/a
Ceftriaxone	>32 (16->32)	>32 (32->32)	0 (0)	0 (0)	0 (0)	0 (0)	13 (100)	14 (100)	n/a
Ceftazidime	>128 (64->128)	>128 (64->128)	0 (0)	0 (0)	0 (0)	0 (0)	13 (100)	14 (100)	n/a
Cefepime	>32 (1->32)	16 (≤0.5 ->32)	1 (7.7)	5 (35.7)	1 (7.7)	1 (7.1)	11 (84.6)	8 (57.1)	0.21
Ertapenem	8 (≤0.12->8)	4 (0.25->8)	2 (15.4)	3 (21.4)	0 (0)	0 (0)	11 (84.6)	11 (78.6)	1.00
Doripenem	2 (≤0.12-8)	1 (≤0.12-8)	5 (38.5)	8 (57.1)	3 (23.1)	2 (14.3)	5 (38.5)	4 (28.6)	0.61
Imipenem	2 (≤0.5-8)	1 (≤0.5->64)	5 (38.5)	7 (50.0)	2 (15.4)	1 (7.1)	6 (46.2)	6 (42.9)	0.73
Piperacillin-Tazobactam	>128 (16->128)	>128 (≤4->128)	1 (7.7)	1 (7.1)	1 (7.7)	0 (0)	11 (84.6)	13 (92.9)	0.57
Meropenem	2 (≤0.12->8)	0.5 (≤0.12->8)	5 (38.5)	8 (57.1)	4 (30.8)	0 (0)	4 (30.8)	6 (42.9)	0.08
**Fluoroquinolones**									
Ciprofloxacin	>8 (0.5->8)	3 (≤0.25->8)	0 (0)	6 (42.9)	1 (7.7)	0 (0)	12 (92.3)	8 (57.1)	0.02
Levofloxacin	>8 (0.1->8)	3 (≤0.12->8)	0 (0)	6 (42.9)	1 (7.7)	0 (0)	12 (92.3)	8 (57.1)	0.02
**Tetracyclines**									
Tetracycline	16 (≤2.0->32)	4 (≤2-32)	5 (38.5)	9 (64.3)	1 (7.7)	1 (7.1)	7 (53.8)	4 (28.6)	0.38
Tigecycline	1 (≤0.5->4)	0.76 (≤0.5->4)	11 (84.6)	9 (64.3)	1 (7.7)	4 (28.6)	1 (7.7)	1 (7.1)	0.37
**Miscellaneous**									

Trimethoprim-Sulfamethoxazole	>8 (1->8)	(≤0.5 (≤0.5->8)	2 (15.4)	12 (85.7)	0 (0)	0 (0)	11 (84.6)	2 (14.3)	0.001

* MICs in μg/mL

** Intermediate is equivalent to the category susceptible dose dependent for cefepime

*** Calculated for proportion *mcr-9* positive categorized as S/I/R compared to *mcr-9* negative isolates.

Abbreviation: MIC: minimum inhibitory concentration

Supplementary Table 3: Plasmid contents of Carbapenem-resistant *E. cloacae* complex genomes *by mcr-9 status*

Isolate	Plasmid	Identity (%)	Plasmid Accession
***mcr-9* positive**			
SRR10377147	ColRNAI	100	DQ298019
	IncFIA(HI1)	98.0	AF250878
	IncHI2A	100	BX664015
	pKPC-CAV1193	98.7	CP013325
SRR3467253	IncHI2A	100	BX664015
SRR4035127	IncHI2A	99.5	BX664015
	IncFII(pECLA)	96.8	CP001919
	IncFIB(pECLA)	99.5	CP001919
SRR4035132	IncHI2A	100	BX664015
	IncR	100	DQ449578
SRR5666570	Col440I	83.2	CP023920
	IncFII(pECLA)	96.1	CP001919
	IncHI2A	100	BX664015
	pKPC-CAV1193	98.7	CP013325
SRR5666425	IncHI2A		BX664015
SRR5666426	IncFIA(HI1)	98.0	AF250878
	IncHI2A	100	BX664015
	pKPC-CAV1193	98.7	CP013325
SRR5666432	IncFIB(pB171)	91.2	AB024946

	IncFII(SARC14)	94.8	JQ418540
	IncHI2A	100	BX664015
	pKPC-CAV1193	98.7	CP013325
SRR10377089	Col440I	83.2	CP023920
	Col440II	84.6	CP023921
	IncHI2A	100	BX664015
	IncX5	99.7	MF062700
SRR10377142	IncFIA(HI1)	98.0	AF250878
	IncFIB(pECLA)	100	CP001919
	IncFII(pECLA)	100	CP001919
	IncHI2A	100	BX664015
SRR10377154	Col440II	83.9	CP023921
	IncFIB(pECLA)	100	CP001919
	IncFII(pECLA)	100	CP001919
SRR12902635	IncFIA(HI1)	98.0	AF250878
	IncFIB(pECLA)	100	CP001919
	IncFII(pECLA)	100	CP001919
	IncHI2A	100	BX664015
	pKPC-CAV1193	98.7	CP013325
SRR12902473	IncFIA(HI1)	97.8	AF250878
	IncFIB(pECLA)	100	CP001919
	IncFII(pECLA)	100	CP001919
	IncHI2A	100	BX664015
	pKPC-CAV1193	98.7	CP013325
SRR6674815	Col440II	84.3	CP023921
	IncFIB(pECLA)	100	CP001919

	IncFII(pECLA)	99.6	CP001919
	IncHI2A	100	BX664015
	IncN	99.6	AY046276
	IncR	100	DQ449578
SRR6674819	Col(pHAD28)		
	Col440II	90.8	CP023921
	IncFIB(K)	84.8	JN233704
	IncFII(pECLA)	99.5	CP001919
	IncHI2A	100	BX664015
	IncQ2	94.2	FJ696404
	IncR	100	DQ449578
SRR6674864	IncHI2A	100	BX664015
	pKPC-CAV1193	100	CP013325
***mcr-9* negative**			
SRR3996257	Col440II	84.4	CP023921
	IncX3	100	JN247852
SRR4035128	n/a		
SRR4035129	Col440II	84.6	
	pKPC-CAV1193	100	CP013325
SRR4035130	n/a		
SRR4035131	n/a		
SRR12902528	Col440II	84.4	CP023921
	IncFII(pECLA)	99.9	CP001919
SRR5666492	IncFIB(pECLA)	100	CP001919
	IncFIB(pENTE01)	81.1	CP000654
	IncFII(pECLA)	100	CP001919
	IncHI1A(NDM-CIT)	100	JX182975

	IncHI1B(pNDM-CIT)	99.1	JX182975
SRR10377150	Col440II	84.4	CP023921
	IncFIB(pB171)	91.2	AB024946
	IncFIB(pHCM2)	97.5	AL513384
	IncFII(SARC14)	94.8	JQ418540
	pKPC-CAV1193	98.7	CP013325
SRR10377039	IncX5	100	
	pKPC-CAV1193	100	CP013325
SRR10377042	IncFIA(HI1)	97.8	AF250878
	IncFIB(pECLA)	100	CP001919
	IncFII(pECLA)	100	CP001919
	IncHI2A	100	BX664015
SRR10377155	IncFII(pECLA)	97.5	
	IncR	94.0	
SRR12902580	IncFIB(pECLA)	100	CP001919
	IncFII(pECLA)	100	CP001919
SRR12902479	IncFIB(pECLA)	100	CP001919
	IncFII(pECLA)	100	CP001919
SRR12902477	IncFIB(pHCM2)	97.3	AL513384
	IncFIB(pQil)	100	JN233705
SRR3996257	IncX3	100	JN247852
SRR6674810	IncN	99.8	AY046276
SRR6674817	IncFIB(pECLA)	100	CP001919
	IncFIB(pENTE01)	80.7	CP000654
	IncFIB(pQil)	100	JN233705
	IncFII(pECLA)	97.9	CP001919
	IncQ2	81.1	FJ696404

	pKPC-CAV1193	100	CP013325
SRR6674821	IncFIB(pECLA)	100	CP001919
	IncFIB(pENTE01)	80.7	CP000654
	IncFIB(pQil)	100	JN233705
	IncFII(pECLA)	99.9	CP001919
	IncQ2	81.1	FJ696404
	pKPC-CAV1193	100	CP013325
SRR6674823	IncFIB(pENTAS01)	93.2	CP003027
	IncFII(SARC14)	99.5	JQ418540
SRR6674824	Col440II	84.0	CP023921
	IncFIB(pECLA)	100	CP001919
	IncFII(pECLA)	99.6	CP001919
	IncN	99.8	AY046276
	IncR	100	DQ449578
SRR6674865	IncN	99.8	AY046276

Supplementary Table 4: The core genome pair-wise SNP distance for cluster of E. cloacae complex genomes from reference 17 (n=13)

	DRX055644	DRX055645	DRX055646	DRX055647	DRX055648	DRX055652	DRX055653	DRX055654	DRX055655	DRX055656	DRX055657	DRX055658	DRX055660
DRX055644	0	2	5	3	2	2	2	5	5	29	2	95	0
DRX055645	2	0	3	1	2	5	2	5	5	29	0	95	2
DRX055646	5	3	0	4	5	8	5	8	8	32	3	98	5
DRX055647	3	1	4	0	3	6	3	6	6	30	1	96	3
DRX055648	2	2	5	3	0	3	0	3	3	27	2	93	2
DRX055652	2	5	8	6	3	0	3	5	6	29	5	95	2
DRX055653	2	2	5	3	0	3	0	3	3	27	2	93	2
DRX055654	5	5	8	6	3	5	3	0	6	30	5	96	5
DRX055655	5	5	8	6	3	6	3	6	0	30	5	90	5
DRX055656	29	29	32	30	27	29	27	30	30	0	29	119	29
DRX055657	2	0	3	1	2	5	2	5	5	29	0	95	2
DRX055658	95	95	98	96	93	95	93	96	90	119	95	0	95
DRX055660	0	2	5	3	2	2	2	5	5	29	2	95	0

The core genome pair-wise SNP distance for each sample is also calculated with snp-dists (48)

Supplementary Table 5: Sample and project accession numbers for *E. cloacae complex* genomes were included in analysis

Sample Accession	Study Accession	Instrument Model	Read Count	Base Count
DRX055644	PRJDB4867	Illumina HiSeq 2500	1780061	445015250
DRX055645	PRJDB4867	Illumina HiSeq 2500	2489022	622255500
DRX055646	PRJDB4867	Illumina HiSeq 2500	1772331	443082750
DRX055647	PRJDB4867	Illumina HiSeq 2500	1807515	451878750
DRX055648	PRJDB4867	Illumina HiSeq 2500	3198149	799537250
DRX055652	PRJDB4867	Illumina HiSeq 2500	1099396	274849000
DRX055653	PRJDB4867	Illumina HiSeq 2500	2443879	610969750
DRX055655	PRJDB4867	Illumina HiSeq 2500	1972856	493214000
DRX055656	PRJDB4867	Illumina HiSeq 2500	1651643	412910750
DRX055657	PRJDB4867	Illumina HiSeq 2500	1648279	412069750
DRX055658	PRJDB4867	Illumina HiSeq 2500	1412999	353249750
DRX055660	PRJDB4867	Illumina HiSeq 2500	1884659	471164750
DRX055661	PRJDB4867	Illumina HiSeq 2500	1732167	433041750
DRX055666	PRJDB4867	Illumina HiSeq 2500	2287372	571843000
DRX055672	PRJDB4867	Illumina HiSeq 2500	2107723	526930750
DRX076672	PRJDB5422	Illumina MiSeq	3996830	1365141966
DRX076673	PRJDB5422	Illumina MiSeq	351994	199762186
DRX115059	PRJDB5929	Illumina HiSeq 3000	4939350	1491683700
DRX115060	PRJDB5929	Illumina HiSeq 3000	3787222	1143741044
DRX115061	PRJDB5929	Illumina HiSeq 3000	3296176	995445152
DRX115062	PRJDB5929	Illumina HiSeq 3000	4061813	1226667526
DRX115063	PRJDB5929	Illumina HiSeq 3000	4700154	1419446508
DRX115064	PRJDB5929	Illumina HiSeq 3000	4406404	1330734008
DRX115065	PRJDB5929	Illumina HiSeq 3000	5391331	1628181962
DRX115066	PRJDB5929	Illumina HiSeq 3000	4657379	1406528458
DRX115313	PRJDB5929	Illumina HiSeq 3000	2816543	850595986
DRX115330	PRJDB5929	Illumina HiSeq 3000	2434702	735280004
DRX115331	PRJDB5929	Illumina HiSeq 3000	2503879	756171458
DRX115352	PRJDB5929	Illumina HiSeq 3000	2584489	780515678
DRX115353	PRJDB5929	Illumina HiSeq 3000	2035809	614814318
DRX115373	PRJDB5929	Illumina HiSeq 3000	2698997	815097094
DRX115376	PRJDB5929	Illumina HiSeq 3000	2112440	637956880
DRX115410	PRJDB5929	Illumina HiSeq 3000	5713464	1725466128
DRX117786	PRJDB6407	Illumina MiSeq	4109736	1241140272

DRX117793	PRJDB6407	Illumina MiSeq	4415553	1333497006
DRX117798	PRJDB6407	Illumina MiSeq	3777259	1140732218
DRX117799	PRJDB6407	Illumina MiSeq	3960589	1196097878
DRX117803	PRJDB6407	Illumina MiSeq	4141233	1250652366
DRX117806	PRJDB6407	Illumina MiSeq	4307043	1300726986
DRX117827	PRJDB6407	Illumina MiSeq	4280611	1292744522
DRX117832	PRJDB6407	Illumina MiSeq	4118903	1243908706
DRX117837	PRJDB6407	Illumina MiSeq	4084292	1233456184
DRX117839	PRJDB6407	Illumina MiSeq	7688360	2321884720
DRX141042	PRJDB5929	Illumina HiSeq 3000	4333756	1308794312
DRX141103	PRJDB5929	Illumina HiSeq 3000	3893751	1175912802
DRX141110	PRJDB5929	Illumina HiSeq 3000	3519759	1062967218
DRX141111	PRJDB5929	Illumina HiSeq 3000	3720050	1123455100
DRX141123	PRJDB5929	Illumina HiSeq 3000	3504633	1058399166
DRX141179	PRJDB5929	Illumina HiSeq 3000	3232937	976346974
DRX141217	PRJDB5929	Illumina HiSeq 3000	2653630	801396260
DRX165616	PRJDB5126	Illumina MiSeq	1459298	732567596
DRX165617	PRJDB5126	Illumina MiSeq	1597518	801954036
DRX165618	PRJDB5126	Illumina MiSeq	1429495	717606490
DRX165619	PRJDB5126	Illumina MiSeq	1230933	617928366
DRX165620	PRJDB5126	Illumina MiSeq	1082942	543636884
DRX165621	PRJDB5126	Illumina MiSeq	1583564	794949128
DRX165622	PRJDB5126	Illumina MiSeq	1575783	791043066
DRX168888	PRJDB8311	HiSeq X Ten	1255235	376570500
DRX168890	PRJDB8311	HiSeq X Ten	1287025	386107500
DRX168891	PRJDB8311	HiSeq X Ten	1387145	416143500
DRX168893	PRJDB8311	HiSeq X Ten	1095205	328561500
DRX168898	PRJDB8311	HiSeq X Ten	1332417	399725100
DRX168901	PRJDB8311	HiSeq X Ten	994753	298425900
DRX168918	PRJDB8311	HiSeq X Ten	1177746	353323800
DRX168920	PRJDB8311	HiSeq X Ten	1136989	341096700
DRX168927	PRJDB8311	HiSeq X Ten	1315896	394768800
DRX168929	PRJDB8311	HiSeq X Ten	1359012	407703600
DRX168935	PRJDB8311	HiSeq X Ten	1199252	359775600
DRX168939	PRJDB8311	HiSeq X Ten	1444447	433334100
DRX168940	PRJDB8311	HiSeq X Ten	1450169	435050700
DRX168941	PRJDB8311	HiSeq X Ten	1293393	388017900
DRX168944	PRJDB8311	HiSeq X Ten	1291997	387599100
DRX168946	PRJDB8311	HiSeq X Ten	1347615	404284500
DRX168948	PRJDB8311	HiSeq X Ten	1157130	347139000

DRX168957	PRJDB8311	HiSeq X Ten	1017134	305140200
DRX168960	PRJDB8311	HiSeq X Ten	1315646	394693800
DRX168963	PRJDB8311	HiSeq X Ten	1306873	392061900
DRX168966	PRJDB8311	HiSeq X Ten	1237180	371154000
DRX168969	PRJDB8311	HiSeq X Ten	1224704	367411200
DRX168972	PRJDB8311	HiSeq X Ten	1547780	464334000
DRX168975	PRJDB8311	HiSeq X Ten	1146999	344099700
DRX168977	PRJDB8311	HiSeq X Ten	1396558	418967400
DRX189420	PRJDB9027	Illumina MiSeq	709964	395811769
DRX189637	PRJDB6962	NextSeq 500	2490184	683827922
DRX189641	PRJDB6962	NextSeq 500	11070687	2966235620
DRX189668	PRJDB6962	NextSeq 500	1448462	432277554
DRX189771	PRJDB6962	NextSeq 500	1023184	292848006
DRX189773	PRJDB6962	NextSeq 500	3332772	992225296
DRX189852	PRJDB6962	NextSeq 500	900442	259605534
DRX189853	PRJDB6962	NextSeq 500	1154874	338358770
DRX189855	PRJDB6962	NextSeq 500	1672979	485806200
DRX189857	PRJDB6962	NextSeq 500	990451	275152008
DRX189883	PRJDB6962	NextSeq 500	1319197	395090411
DRX189909	PRJDB6962	NextSeq 500	745414	215572523
DRX190187	PRJDB9036	NextSeq 500	833785	243641576
DRX190192	PRJDB9036	NextSeq 500	3576132	1039952378
DRX190205	PRJDB9036	NextSeq 500	1573402	467574583
DRX231979	PRJDB10450	Illumina MiSeq	1058879	499604439
DRX241994	PRJDB6565	Illumina MiSeq	1196533	600659566
DRX241996	PRJDB6565	Illumina MiSeq	1515252	760656504
DRX241997	PRJDB6565	Illumina MiSeq	1431156	718440312
DRX264865	PRJDB5126	Illumina MiSeq	1707255	857042010
DRX264874	PRJDB5126	Illumina MiSeq	1647358	826973716
DRX313429	PRJDB11927	Illumina iSeq 100	856820	248054738
DRX323930	PRJDB12737	Illumina MiSeq	369190	220447785
DRX323931	PRJDB12737	Illumina MiSeq	562106	334440969
DRX323932	PRJDB12737	Illumina MiSeq	596674	354430068
DRX323933	PRJDB12737	Illumina MiSeq	556826	329694445
DRX323934	PRJDB12737	Illumina MiSeq	428701	254593750
DRX323935	PRJDB12737	Illumina MiSeq	416222	248680297
DRX323936	PRJDB12737	Illumina MiSeq	235691	141143931
DRX323937	PRJDB12737	Illumina MiSeq	476949	284250124
DRX323938	PRJDB12737	Illumina MiSeq	548465	327293408
DRX323939	PRJDB12737	Illumina MiSeq	491195	292610786

DRX323941	PRJDB12737	Illumina MiSeq	487405	265977346
DRX323942	PRJDB12737	Illumina MiSeq	668484	389276884
DRX323943	PRJDB12737	Illumina MiSeq	405354	233101454
ERX1094431	PRJEB6891	Illumina HiSeq 2000	6929308	866163500
ERX1180282	PRJEB10263	Illumina MiSeq	2701902	405285300
ERX1423194	PRJEB13304	Illumina MiSeq	1275829	623621719
ERX1587240	PRJEB12699	Illumina HiSeq 2000	5704990	713123750
ERX1587241	PRJEB12699	Illumina HiSeq 2000	5400778	675097250
ERX1587242	PRJEB12699	Illumina HiSeq 2000	5355564	669445500
ERX1587243	PRJEB12699	Illumina HiSeq 2000	5534976	691872000
ERX1587244	PRJEB12699	Illumina HiSeq 2000	5514592	689324000
ERX1587245	PRJEB12699	Illumina HiSeq 2000	5482942	685367750
ERX1587246	PRJEB12699	Illumina HiSeq 2000	5638500	704812500
ERX1587247	PRJEB12699	Illumina HiSeq 2000	6370602	796325250
ERX1587248	PRJEB12699	Illumina HiSeq 2000	5977350	747168750
ERX1587249	PRJEB12699	Illumina HiSeq 2000	5089368	636171000
ERX1587250	PRJEB12699	Illumina HiSeq 2000	5748210	718526250
ERX1587251	PRJEB12699	Illumina HiSeq 2000	5776258	722032250
ERX1587252	PRJEB12699	Illumina HiSeq 2000	6209186	776148250
ERX1587253	PRJEB12699	Illumina HiSeq 2000	5971830	746478750
ERX1587254	PRJEB12699	Illumina HiSeq 2000	5273644	659205500
ERX1587255	PRJEB12699	Illumina HiSeq 2000	7023114	877889250
ERX1587256	PRJEB12699	Illumina HiSeq 2000	5875414	734426750
ERX1587257	PRJEB12699	Illumina HiSeq 2000	6284894	785611750
ERX1587258	PRJEB12699	Illumina HiSeq 2000	5249396	656174500
ERX1656325	PRJEB12888	Illumina HiSeq 2000	2785376	348172000
ERX1656369	PRJEB12888	Illumina HiSeq 2000	2506140	313267500
ERX1656371	PRJEB12888	Illumina HiSeq 2000	2425090	303136250
ERX1656385	PRJEB12888	Illumina HiSeq 2000	2415904	301988000
ERX1656387	PRJEB12888	Illumina HiSeq 2000	2402082	300260250
ERX1656418	PRJEB12888	Illumina HiSeq 2000	2917994	364749250
ERX1656424	PRJEB12888	Illumina HiSeq 2000	2452324	306540500
ERX1656435	PRJEB12888	Illumina HiSeq 2000	2526024	315753000
ERX1656442	PRJEB12888	Illumina HiSeq 2000	2590458	323807250
ERX1656451	PRJEB12888	Illumina HiSeq 2000	2451116	306389500
ERX1656458	PRJEB12888	Illumina HiSeq 2000	2497272	312159000
ERX1656474	PRJEB12888	Illumina HiSeq 2000	2577348	322168500
ERX1665900	PRJEB12888	Illumina HiSeq 2000	2738716	342339500
ERX1665901	PRJEB12888	Illumina HiSeq 2000	2831572	353946500
ERX1665902	PRJEB12888	Illumina HiSeq 2000	3080780	385097500

ERX1665942	PRJEB12888	Illumina HiSeq 2000	2788608	348576000
ERX1665943	PRJEB12888	Illumina HiSeq 2000	2906780	363347500
ERX1665944	PRJEB12888	Illumina HiSeq 2000	2973282	371660250
ERX1665947	PRJEB12888	Illumina HiSeq 2000	2499398	312424750
ERX1665951	PRJEB12888	Illumina HiSeq 2000	2477888	309736000
ERX1665952	PRJEB12888	Illumina HiSeq 2000	2634186	329273250
ERX1665954	PRJEB12888	Illumina HiSeq 2000	2741964	342745500
ERX1665955	PRJEB12888	Illumina HiSeq 2000	2510804	313850500
ERX1665959	PRJEB12888	Illumina HiSeq 2000	3042824	380353000
ERX1665961	PRJEB12888	Illumina HiSeq 2000	2359454	294931750
ERX1665963	PRJEB12888	Illumina HiSeq 2000	2277182	284647750
ERX1665964	PRJEB12888	Illumina HiSeq 2000	2538262	317282750
ERX1665965	PRJEB12888	Illumina HiSeq 2000	2668998	333624750
ERX1665966	PRJEB12888	Illumina HiSeq 2000	2448656	306082000
ERX1665967	PRJEB12888	Illumina HiSeq 2000	2525108	315638500
ERX1665968	PRJEB12888	Illumina HiSeq 2000	2517004	314625500
ERX1665969	PRJEB12888	Illumina HiSeq 2000	2320834	290104250
ERX1665970	PRJEB12888	Illumina HiSeq 2000	2566036	320754500
ERX1665971	PRJEB12888	Illumina HiSeq 2000	2286112	285764000
ERX1665976	PRJEB12888	Illumina HiSeq 2000	2571282	321410250
ERX1666004	PRJEB12888	Illumina HiSeq 2000	2797538	349692250
ERX1666005	PRJEB12888	Illumina HiSeq 2000	2699806	337475750
ERX1666007	PRJEB12888	Illumina HiSeq 2000	2759380	344922500
ERX1666008	PRJEB12888	Illumina HiSeq 2000	2693750	336718750
ERX1666016	PRJEB12888	Illumina HiSeq 2000	2734918	341864750
ERX1666027	PRJEB12888	Illumina HiSeq 2000	2974808	371851000
ERX1666033	PRJEB12888	Illumina HiSeq 2000	3082800	385350000
ERX168339	PRJEB3353	Illumina MiSeq	1059441	317832300
ERX168340	PRJEB3353	Illumina MiSeq	963357	289007100
ERX168341	PRJEB3353	Illumina MiSeq	1155077	346523100
ERX168342	PRJEB3353	Illumina MiSeq	632913	189873900
ERX168343	PRJEB3353	Illumina MiSeq	527615	158284500
ERX168344	PRJEB3353	Illumina MiSeq	884030	265209000
ERX168345	PRJEB3353	Illumina MiSeq	394359	118307700
ERX168346	PRJEB3353	Illumina MiSeq	1048216	314464800
ERX168347	PRJEB3353	Illumina MiSeq	985476	295642800
ERX1686613	PRJEB15226	Illumina MiSeq	966042	433149897
ERX1710927	PRJEB12888	Illumina HiSeq 2000	2136860	267107500
ERX1710928	PRJEB12888	Illumina HiSeq 2000	2050748	256343500
ERX1710931	PRJEB12888	Illumina HiSeq 2000	2001680	250210000

ERX1710932	PRJEB12888	Illumina HiSeq 2000	1879108	234888500
ERX1710960	PRJEB12888	Illumina HiSeq 2000	2000360	250045000
ERX1710963	PRJEB12888	Illumina HiSeq 2000	2097518	262189750
ERX1710964	PRJEB12888	Illumina HiSeq 2000	1948754	243594250
ERX1710980	PRJEB12888	Illumina HiSeq 2000	1992200	249025000
ERX1711011	PRJEB12888	Illumina HiSeq 2000	2188404	273550500
ERX1711016	PRJEB12888	Illumina HiSeq 2000	1998576	249822000
ERX1711029	PRJEB12888	Illumina HiSeq 2000	2047394	255924250
ERX1711033	PRJEB12888	Illumina HiSeq 2000	1874906	234363250
ERX1938500	PRJEB19677	Illumina MiSeq	971442	268918505
ERX1938501	PRJEB19677	Illumina MiSeq	684833	196890706
ERX1938502	PRJEB19677	Illumina MiSeq	584923	163937299
ERX1938503	PRJEB19677	Illumina MiSeq	726700	199778727
ERX1938504	PRJEB19677	Illumina MiSeq	888517	245727920
ERX1938506	PRJEB19677	Illumina MiSeq	270593	79129790
ERX1951637	PRJEB8666	Illumina HiSeq 2000	2420812	302601500
ERX1951638	PRJEB8666	Illumina HiSeq 2000	2498776	312347000
ERX1951986	PRJEB8666	Illumina HiSeq 2000	2682456	335307000
ERX1951989	PRJEB8666	Illumina HiSeq 2000	3303366	412920750
ERX1951999	PRJEB8666	Illumina HiSeq 2000	2347832	293479000
ERX1952011	PRJEB8666	Illumina HiSeq 2000	2725882	340735250
ERX1952014	PRJEB8666	Illumina HiSeq 2000	2584512	323064000
ERX1952029	PRJEB8666	Illumina HiSeq 2000	162894	20361750
ERX1952057	PRJEB8666	Illumina HiSeq 2000	2612968	326621000
ERX1952058	PRJEB8666	Illumina HiSeq 2000	2607846	325980750
ERX2000466	PRJEB8104	Illumina HiSeq 2500	7440382	930047750
ERX2016430	PRJEB19777	Illumina MiSeq	2308162	806849452
ERX2049035	PRJEB20875	NextSeq 500	1183052	323152151
ERX2049052	PRJEB20875	NextSeq 500	1044743	499215804
ERX2164577	PRJEB22264	NextSeq 500	3076477	860079439
ERX2164578	PRJEB22264	NextSeq 500	2740987	771033898
ERX2164579	PRJEB22264	NextSeq 500	2769438	780650130
ERX2164580	PRJEB22264	NextSeq 500	3094521	885731029
ERX2164581	PRJEB22264	NextSeq 500	2893344	813731379
ERX2164582	PRJEB22264	NextSeq 500	3056589	855805863
ERX2164583	PRJEB22264	NextSeq 500	2759735	778131723
ERX2169928	PRJEB22119	Illumina MiSeq	1132982	601381821
ERX2169929	PRJEB22119	Illumina MiSeq	960666	284698905
ERX2169930	PRJEB22119	Illumina MiSeq	676502	308999675
ERX2169931	PRJEB22119	Illumina MiSeq	982287	457205139

ERX2169932	PRJEB22119	Illumina MiSeq	350187	198260410
ERX2169933	PRJEB22119	Illumina MiSeq	821379	397615070
ERX2169934	PRJEB22119	Illumina MiSeq	879858	412163424
ERX2169935	PRJEB22119	Illumina MiSeq	580846	247966630
ERX2169936	PRJEB22119	Illumina MiSeq	533752	212628818
ERX2169937	PRJEB22119	Illumina MiSeq	897063	309097812
ERX2169938	PRJEB22119	Illumina MiSeq	583696	324956178
ERX2181402	PRJEB22264	NextSeq 500	3355854	946584763
ERX2181403	PRJEB22264	NextSeq 500	2985759	828477159
ERX2181404	PRJEB22264	NextSeq 500	2974011	835394361
ERX2181405	PRJEB22264	NextSeq 500	2827547	796120648
ERX2181406	PRJEB22264	NextSeq 500	2635224	742896632
ERX2181407	PRJEB22264	NextSeq 500	2690415	759741142
ERX2181408	PRJEB22264	NextSeq 500	2529330	717960553
ERX2181409	PRJEB22264	NextSeq 500	2775829	777673848
ERX2181410	PRJEB22264	NextSeq 500	2880832	802331428
ERX2181411	PRJEB22264	NextSeq 500	2525329	701978147
ERX2274529	PRJEB23845	Illumina HiSeq 2000	2308983	461796600
ERX2290109	PRJEB21277	Illumina HiSeq 2500	1485069	445520700
ERX2290111	PRJEB21277	Illumina HiSeq 2500	1671576	501472800
ERX2290143	PRJEB21277	Illumina HiSeq 2500	1585789	475736700
ERX2290161	PRJEB21277	Illumina HiSeq 2500	1875775	562732500
ERX2290162	PRJEB21277	Illumina HiSeq 2500	1615085	484525500
ERX2290165	PRJEB21277	Illumina HiSeq 2500	1711762	513528600
ERX2290172	PRJEB21277	Illumina HiSeq 2500	1737983	521394900
ERX2290184	PRJEB21277	Illumina HiSeq 2500	1395710	418713000
ERX2290207	PRJEB21277	Illumina HiSeq 2500	1423986	427195800
ERX2290227	PRJEB21277	Illumina HiSeq 2500	1512346	453703800
ERX2290237	PRJEB21277	Illumina HiSeq 2500	1576586	472975800
ERX2290253	PRJEB21277	Illumina HiSeq 2500	1677009	503102700
ERX2290271	PRJEB21277	Illumina HiSeq 2500	1675062	502518600
ERX2290278	PRJEB21277	Illumina HiSeq 2500	1453489	436046700
ERX2290282	PRJEB21277	Illumina HiSeq 2500	1379821	413946300
ERX2438396	PRJEB19322	HiSeq X Ten	3278366	495033266
ERX2438397	PRJEB19322	HiSeq X Ten	2842874	429273974
ERX2438398	PRJEB19322	HiSeq X Ten	3159336	477059736
ERX2438399	PRJEB19322	HiSeq X Ten	3045928	459935128
ERX2438400	PRJEB19322	HiSeq X Ten	2110354	318663454
ERX2438401	PRJEB19322	HiSeq X Ten	2429412	366841212
ERX2438402	PRJEB19322	HiSeq X Ten	2767600	417907600

ERX2438403	PRJEB19322	HiSeq X Ten	2805250	423592750
ERX2438404	PRJEB19322	HiSeq X Ten	2911414	439623514
ERX2438405	PRJEB19322	HiSeq X Ten	3767580	568904580
ERX2438406	PRJEB19322	HiSeq X Ten	1540698	232645398
ERX2438407	PRJEB19322	HiSeq X Ten	1417710	214074210
ERX2438408	PRJEB19322	HiSeq X Ten	2201370	332406870
ERX2438409	PRJEB19322	HiSeq X Ten	2051444	309768044
ERX2438410	PRJEB19322	HiSeq X Ten	2820662	425919962
ERX2438411	PRJEB19322	HiSeq X Ten	2430384	366987984
ERX2438412	PRJEB19322	HiSeq X Ten	2201654	332449754
ERX2438413	PRJEB19322	HiSeq X Ten	3097444	467714044
ERX2438414	PRJEB19322	HiSeq X Ten	2529864	382009464
ERX2438415	PRJEB19322	HiSeq X Ten	2131118	321798818
ERX2438416	PRJEB19322	HiSeq X Ten	2409894	363893994
ERX2438417	PRJEB19322	HiSeq X Ten	2250846	339877746
ERX2438418	PRJEB19322	HiSeq X Ten	3263000	492713000
ERX2438419	PRJEB19322	HiSeq X Ten	2458570	371244070
ERX2438420	PRJEB19322	HiSeq X Ten	2379856	359358256
ERX2438421	PRJEB19322	HiSeq X Ten	2346340	354297340
ERX2438422	PRJEB19322	HiSeq X Ten	2391424	361105024
ERX2438423	PRJEB19322	HiSeq X Ten	2881590	435120090
ERX2438424	PRJEB19322	HiSeq X Ten	2174628	328368828
ERX2438425	PRJEB19322	HiSeq X Ten	2408684	363711284
ERX2438426	PRJEB19322	HiSeq X Ten	2645870	399526370
ERX2438427	PRJEB19322	HiSeq X Ten	2446562	369430862
ERX2438428	PRJEB19322	HiSeq X Ten	3298048	498005248
ERX2438429	PRJEB19322	HiSeq X Ten	2652344	400503944
ERX2438430	PRJEB19322	HiSeq X Ten	2294554	346477654
ERX2438431	PRJEB19322	HiSeq X Ten	2351756	355115156
ERX2438432	PRJEB19322	HiSeq X Ten	2231138	336901838
ERX2438433	PRJEB19322	HiSeq X Ten	2742744	414154344
ERX2438434	PRJEB19322	HiSeq X Ten	2656316	401103716
ERX2438435	PRJEB19322	HiSeq X Ten	2276224	343709824
ERX2438436	PRJEB19322	HiSeq X Ten	2288562	345572862
ERX2438437	PRJEB19322	HiSeq X Ten	2136252	322574052
ERX2438438	PRJEB19322	HiSeq X Ten	2272198	343101898
ERX2438439	PRJEB19322	HiSeq X Ten	2482220	374815220
ERX2438440	PRJEB19322	HiSeq X Ten	2884136	435504536
ERX2438441	PRJEB19322	HiSeq X Ten	2620696	395725096
ERX2438442	PRJEB19322	HiSeq X Ten	2473978	373570678

ERX2438443	PRJEB19322	HiSeq X Ten	2243430	338757930
ERX2438444	PRJEB19322	HiSeq X Ten	2228194	336457294
ERX2438445	PRJEB19322	HiSeq X Ten	2969044	448325644
ERX2438446	PRJEB19322	HiSeq X Ten	2352520	355230520
ERX2438447	PRJEB19322	HiSeq X Ten	2374166	358499066
ERX2438448	PRJEB19322	HiSeq X Ten	2702948	408145148
ERX2438449	PRJEB19322	HiSeq X Ten	3094610	467286110
ERX2438450	PRJEB19322	HiSeq X Ten	2588996	390938396
ERX2438451	PRJEB19322	HiSeq X Ten	2395974	361792074
ERX2438452	PRJEB19322	HiSeq X Ten	2476252	373914052
ERX2438453	PRJEB19322	HiSeq X Ten	2227076	336288476
ERX2438454	PRJEB19322	HiSeq X Ten	2329294	351723394
ERX2438455	PRJEB19322	HiSeq X Ten	2725830	411600330
ERX2438456	PRJEB19322	HiSeq X Ten	2754152	415876952
ERX2438457	PRJEB19322	HiSeq X Ten	3378368	510133568
ERX2438458	PRJEB19322	HiSeq X Ten	3031104	457696704
ERX2438459	PRJEB19322	HiSeq X Ten	2472376	373328776
ERX2438460	PRJEB19322	HiSeq X Ten	2307794	348476894
ERX2438461	PRJEB19322	HiSeq X Ten	2498956	377342356
ERX2438462	PRJEB19322	HiSeq X Ten	2194966	331439866
ERX2516633	PRJEB19322	HiSeq X Ten	2916726	440425626
ERX2516707	PRJEB19322	HiSeq X Ten	2120256	320158656
ERX2516708	PRJEB19322	HiSeq X Ten	2194572	331380372
ERX2516709	PRJEB19322	HiSeq X Ten	2452914	370390014
ERX2516710	PRJEB19322	HiSeq X Ten	2619484	395542084
ERX2516711	PRJEB19322	HiSeq X Ten	2759418	416672118
ERX2516712	PRJEB19322	HiSeq X Ten	2489464	375909064
ERX2516713	PRJEB19322	HiSeq X Ten	2538354	383291454
ERX2516714	PRJEB19322	HiSeq X Ten	2264540	341945540
ERX2516715	PRJEB19322	HiSeq X Ten	2086188	315014388
ERX2669114	PRJEB27344	Illumina HiSeq 2500	1202585	233778888
ERX2669115	PRJEB27344	Illumina HiSeq 2500	1339070	261020384
ERX2669116	PRJEB27344	Illumina HiSeq 2500	1199754	233564681
ERX2669117	PRJEB27344	Illumina HiSeq 2500	1134176	217623802
ERX2669118	PRJEB27344	Illumina HiSeq 2500	1076089	209784897
ERX2669119	PRJEB27344	Illumina HiSeq 2500	3527535	692091673
ERX2696319	PRJEB14854	Illumina MiSeq	1702696	255404400
ERX2696320	PRJEB14854	Illumina MiSeq	1607114	241067100
ERX2733219	PRJEB20809	HiSeq X Ten	1841560	278075560
ERX2733220	PRJEB20809	HiSeq X Ten	1774768	267989968

ERX2733221	PRJEB20809	HiSeq X Ten	1932058	291740758
ERX2733222	PRJEB20809	HiSeq X Ten	1974042	298080342
ERX2733223	PRJEB20809	HiSeq X Ten	1877394	283486494
ERX2733224	PRJEB20809	HiSeq X Ten	1793768	270858968
ERX2733225	PRJEB20809	HiSeq X Ten	1574772	237790572
ERX2733226	PRJEB20809	HiSeq X Ten	1738620	262531620
ERX2733227	PRJEB20809	HiSeq X Ten	1793976	270890376
ERX2733228	PRJEB20809	HiSeq X Ten	1979518	298907218
ERX2733229	PRJEB20809	HiSeq X Ten	1703348	257205548
ERX2733230	PRJEB20809	HiSeq X Ten	1844726	278553626
ERX2733231	PRJEB20809	HiSeq X Ten	1964486	296637386
ERX2733232	PRJEB20809	HiSeq X Ten	1944616	293637016
ERX2733233	PRJEB20809	HiSeq X Ten	1731402	261441702
ERX2733234	PRJEB20809	HiSeq X Ten	1836238	277271938
ERX2733235	PRJEB20809	HiSeq X Ten	1609112	242975912
ERX2733236	PRJEB20809	HiSeq X Ten	1668110	251884610
ERX2733237	PRJEB20809	HiSeq X Ten	1867012	281918812
ERX284790	PRJEB1271	Illumina HiSeq 2000	2665336	533067200
ERX284794	PRJEB1271	Illumina HiSeq 2000	2535769	507153800
ERX284797	PRJEB1271	Illumina HiSeq 2000	2065132	413026400
ERX284798	PRJEB1271	Illumina HiSeq 2000	2370243	474048600
ERX284804	PRJEB1271	Illumina HiSeq 2000	2301169	460233800
ERX284807	PRJEB1271	Illumina HiSeq 2000	2373456	474691200
ERX284837	PRJEB1271	Illumina HiSeq 2000	1686190	337238000
ERX284838	PRJEB1271	Illumina HiSeq 2000	2361062	472212400
ERX287543	PRJEB1271	Illumina HiSeq 2000	2405953	481190600
ERX287544	PRJEB1271	Illumina HiSeq 2000	1964673	392934600
ERX287546	PRJEB1271	Illumina HiSeq 2000	1990939	398187800
ERX287577	PRJEB1271	Illumina HiSeq 2000	2972312	594462400
ERX287584	PRJEB1271	Illumina HiSeq 2000	2385324	477064800
ERX287593	PRJEB1271	Illumina HiSeq 2000	2628347	525669400
ERX287611	PRJEB1271	Illumina HiSeq 2000	2882506	576501200
ERX287641	PRJEB1271	Illumina HiSeq 2000	2486897	497379400
ERX287657	PRJEB1271	Illumina HiSeq 2000	2172817	434563400
ERX287658	PRJEB1271	Illumina HiSeq 2000	2466543	493308600
ERX287660	PRJEB1271	Illumina HiSeq 2000	2325049	465009800
ERX2933845	PRJEB29745	Illumina MiSeq	5804060	2366919833
ERX2986841	PRJEB30118	Illumina MiSeq	693453	323265655
ERX3192745	PRJEB24641	Illumina MiSeq	703902	105585300
ERX3192746	PRJEB24641	Illumina MiSeq	679480	101922000

ERX3192747	PRJEB24641	Illumina MiSeq	796722	119508300
ERX3192748	PRJEB24641	Illumina MiSeq	632860	94929000
ERX3209420	PRJEB23879	HiSeq X Ten	5282822	797706122
ERX3209433	PRJEB23879	HiSeq X Ten	3931284	593623884
ERX3209442	PRJEB23879	HiSeq X Ten	4746842	716773142
ERX3209469	PRJEB23879	HiSeq X Ten	4914712	742121512
ERX3209472	PRJEB23879	HiSeq X Ten	3854596	582043996
ERX3209474	PRJEB23879	HiSeq X Ten	4194076	633305476
ERX3209476	PRJEB23879	HiSeq X Ten	3680844	555807444
ERX3209478	PRJEB23879	HiSeq X Ten	3880320	585928320
ERX3209482	PRJEB23879	HiSeq X Ten	3425718	517283418
ERX3209483	PRJEB23879	HiSeq X Ten	3652112	551468912
ERX3209485	PRJEB23879	HiSeq X Ten	3325394	502134494
ERX3236152	PRJEB30858	Illumina HiSeq 2500	1197080	235095625
ERX3245768	PRJEB23879	HiSeq X Ten	5056398	763516098
ERX3283194	PRJEB31941	Illumina HiSeq 2500	305226	105621584
ERX329036	PRJEB4735	Illumina MiSeq	496487	142168455
ERX3426823	PRJEB22252	HiSeq X Ten	1868268	282108468
ERX3535538	PRJEB34353	Illumina MiSeq	689449	358330556
ERX3535539	PRJEB34353	Illumina MiSeq	604215	331759257
ERX3552073	PRJEB34513	NextSeq 550	2529330	717960553
ERX3565606	PRJEB28400	HiSeq X Ten	3668560	553952560
ERX3565632	PRJEB28400	HiSeq X Ten	4826346	728778246
ERX3565794	PRJEB28400	HiSeq X Ten	4403978	665000678
ERX3565798	PRJEB28400	HiSeq X Ten	3732368	563587568
ERX3565873	PRJEB28400	HiSeq X Ten	3918128	591637328
ERX3580885	PRJEB28400	HiSeq X Ten	4657544	703289144
ERX3580887	PRJEB28400	HiSeq X Ten	4542088	685855288
ERX3580920	PRJEB28400	HiSeq X Ten	3545254	535333354
ERX3581050	PRJEB28400	HiSeq X Ten	3920680	592022680
ERX3581051	PRJEB28400	HiSeq X Ten	4367266	659457166
ERX3581062	PRJEB28400	HiSeq X Ten	5113794	772182894
ERX3581063	PRJEB28400	HiSeq X Ten	5158012	778859812
ERX3581104	PRJEB28400	HiSeq X Ten	4720482	712792782
ERX3581192	PRJEB28400	HiSeq X Ten	3952718	596860418
ERX3581195	PRJEB28400	HiSeq X Ten	4083954	616677054
ERX3591825	PRJEB28400	HiSeq X Ten	3327250	502414750
ERX3591862	PRJEB28400	HiSeq X Ten	3389746	511851646
ERX3635748	PRJEB28400	HiSeq X Ten	4573374	690579474
ERX3635752	PRJEB28400	HiSeq X Ten	5134302	775279602

ERX3635949	PRJEB28400	HiSeq X Ten	3811498	575536198
ERX3647858	PRJEB28400	HiSeq X Ten	4722456	713090856
ERX3647934	PRJEB28400	HiSeq X Ten	4281526	646510426
ERX3647936	PRJEB28400	HiSeq X Ten	4720968	712866168
ERX3647950	PRJEB28400	HiSeq X Ten	3757838	567433538
ERX3648003	PRJEB28400	HiSeq X Ten	4313740	651374740
ERX3715801	PRJEB35685	Illumina HiSeq 2500	2489638	593997570
ERX3715803	PRJEB35685	Illumina HiSeq 2500	1372456	345858912
ERX3715804	PRJEB35685	Illumina HiSeq 2500	1527973	385049196
ERX3715828	PRJEB35685	Illumina HiSeq 2500	1016438	256142376
ERX3715834	PRJEB35685	Illumina HiSeq 2500	1258830	317225160
ERX3715837	PRJEB35685	Illumina HiSeq 2500	1215413	306284076
ERX3715845	PRJEB35685	Illumina HiSeq 2500	1525712	384479424
ERX3715846	PRJEB35685	Illumina HiSeq 2500	4153155	1046595060
ERX3715849	PRJEB35685	Illumina HiSeq 2500	2967128	714124949
ERX3715850	PRJEB35685	Illumina HiSeq 2500	2027960	494470432
ERX3715856	PRJEB35685	Illumina HiSeq 2500	1736428	422952556
ERX3715859	PRJEB35685	Illumina HiSeq 2500	1308786	326489501
ERX3715863	PRJEB35685	Illumina HiSeq 2500	2231320	540157735
ERX3715865	PRJEB35685	Illumina HiSeq 2500	1610809	397829027
ERX3715866	PRJEB35685	Illumina HiSeq 2500	1612726	397173519
ERX3715867	PRJEB35685	Illumina HiSeq 2500	1838737	459505041
ERX3715868	PRJEB35685	Illumina HiSeq 2500	2493743	603952192
ERX3715890	PRJEB35685	Illumina HiSeq 2500	2488485	613475473
ERX3715891	PRJEB35685	Illumina HiSeq 2500	1337946	327806271
ERX3715893	PRJEB35685	Illumina HiSeq 2500	1634362	401596025
ERX3715907	PRJEB35685	Illumina HiSeq 2500	1296078	316545553
ERX3715909	PRJEB35685	Illumina HiSeq 2500	5274508	1235568815
ERX3715913	PRJEB35685	Illumina HiSeq 2500	1976351	483482643
ERX3715915	PRJEB35685	Illumina HiSeq 2500	1045223	257617530
ERX3715921	PRJEB35685	Illumina HiSeq 2500	1771717	433133407
ERX3715932	PRJEB35685	Illumina HiSeq 2500	2504668	622776050
ERX3715934	PRJEB35685	Illumina HiSeq 2500	2602223	650338456
ERX3715944	PRJEB35685	Illumina HiSeq 2500	2287891	557311340
ERX3715947	PRJEB35685	Illumina HiSeq 2500	2001928	496369505
ERX3715956	PRJEB35685	Illumina HiSeq 2500	2231647	551085738
ERX3716008	PRJEB35685	Illumina HiSeq 2500	2421267	605583813
ERX3716015	PRJEB35685	Illumina HiSeq 2500	2907815	727449114
ERX3716023	PRJEB35685	Illumina HiSeq 2500	1923386	458901936
ERX3716048	PRJEB35685	Illumina HiSeq 2500	1509136	359190534

ERX3716052	PRJEB35685	Illumina HiSeq 2500	1885625	448564220
ERX3716070	PRJEB35685	Illumina HiSeq 2500	2108685	517866196
ERX3716084	PRJEB35685	Illumina HiSeq 2500	1188799	296802296
ERX3716085	PRJEB35685	Illumina HiSeq 2500	1673018	411729320
ERX3716094	PRJEB35685	Illumina HiSeq 2500	1769257	428279617
ERX3716106	PRJEB35685	Illumina HiSeq 2500	1915448	469377749
ERX3716118	PRJEB35685	Illumina HiSeq 2500	2195695	528337765
ERX3716119	PRJEB35685	Illumina HiSeq 2500	2400348	571998331
ERX3716142	PRJEB35685	Illumina HiSeq 2500	2327648	554175060
ERX3716169	PRJEB35685	Illumina HiSeq 2500	3321872	796643843
ERX3716313	PRJEB35685	Illumina HiSeq 2500	2006572	481959101
ERX3716339	PRJEB35685	Illumina HiSeq 2500	4279737	1074272462
ERX3716363	PRJEB35685	Illumina HiSeq 2500	1696924	418990245
ERX3716376	PRJEB35685	Illumina HiSeq 2500	1699622	409620160
ERX3716380	PRJEB35685	Illumina HiSeq 2500	2085780	495706617
ERX3716493	PRJEB35685	Illumina HiSeq 2500	2325389	556214251
ERX3716509	PRJEB35685	Illumina HiSeq 2500	2792167	662704883
ERX3716555	PRJEB35685	Illumina HiSeq 2500	1860932	441026965
ERX3716557	PRJEB35685	Illumina HiSeq 2500	1939317	464304804
ERX3716566	PRJEB35685	Illumina HiSeq 2500	2471421	586748508
ERX3716585	PRJEB35685	Illumina HiSeq 2500	2644973	657936803
ERX3716653	PRJEB35685	Illumina HiSeq 2500	2480577	613736589
ERX4015552	PRJEB37711	NextSeq 500	1680245	490399411
ERX4015595	PRJEB37711	NextSeq 500	1579684	464331877
ERX4015666	PRJEB37711	NextSeq 500	1912116	568280985
ERX4015685	PRJEB37711	NextSeq 500	2952236	855357724
ERX4015924	PRJEB37711	NextSeq 500	1039239	305595373
ERX4025284	PRJEB29424	HiSeq X Ten	2399794	362368894
ERX4046611	PRJEB29424	HiSeq X Ten	2626858	396655558
ERX4046720	PRJEB29424	HiSeq X Ten	2441012	368592812
ERX4046772	PRJEB29424	HiSeq X Ten	2460912	371597712
ERX4046977	PRJEB29424	HiSeq X Ten	2512668	379412868
ERX4047020	PRJEB29424	HiSeq X Ten	2455896	370840296
ERX4047473	PRJEB29424	HiSeq X Ten	2289764	345754364
ERX4047482	PRJEB29424	HiSeq X Ten	2314960	349558960
ERX4047486	PRJEB29424	HiSeq X Ten	2488036	375693436
ERX4047587	PRJEB29424	HiSeq X Ten	1854952	280097752
ERX4064048	PRJEB37967	Illumina HiSeq 4000	5678130	1714795260
ERX4064049	PRJEB37967	Illumina HiSeq 4000	5713973	1725619846
ERX4064050	PRJEB37967	Illumina HiSeq 4000	7821431	2362072162

ERX4064051	PRJEB37967	Illumina HiSeq 4000	5784212	1746832024
ERX4106634	PRJEB29424	HiSeq X Ten	2656822	401180122
ERX4316651	PRJEB39112	Illumina NovaSeq 6000	415595	125509690
ERX440121	PRJEB5065	Illumina HiSeq 2000	1908347	381669400
ERX440127	PRJEB5065	Illumina HiSeq 2000	2038430	407686000
ERX4410819	PRJEB39816	Illumina MiSeq	3201335	955991957
ERX4442422	PRJEB32655	HiSeq X Ten	2253738	340314438
ERX4442507	PRJEB32655	HiSeq X Ten	1903096	287367496
ERX4496439	PRJEB40194	Illumina MiSeq	1750193	714492570
ERX4496441	PRJEB40194	Illumina MiSeq	2314300	852922634
ERX452057	PRJEB5065	Illumina HiSeq 2000	1976156	395231200
ERX4623637	PRJEB40571	Illumina MiSeq	1666630	254616958
ERX4623639	PRJEB40571	Illumina MiSeq	2171036	466276427
ERX4652924	PRJEB29739	HiSeq X Ten	2229206	336610106
ERX4785058	PRJEB33565	Illumina MiSeq	1062502	277037643
ERX4785059	PRJEB33565	Illumina MiSeq	469834	130099012
ERX4785064	PRJEB33565	Illumina MiSeq	733410	204197062
ERX4785067	PRJEB33565	Illumina MiSeq	1580480	402573659
ERX4785076	PRJEB33565	Illumina MiSeq	525916	150987781
ERX4785078	PRJEB33565	Illumina MiSeq	977688	264273034
ERX4785106	PRJEB33565	Illumina MiSeq	976220	263546978
ERX4785110	PRJEB33565	Illumina MiSeq	1024546	284328589
ERX4785127	PRJEB33565	Illumina MiSeq	680718	197062012
ERX4795165	PRJEB41940	Illumina HiSeq 2500	5842242	590066442
ERX4795176	PRJEB41940	Illumina HiSeq 2500	5920350	710856669
ERX4795179	PRJEB41940	Illumina HiSeq 2500	4758888	591712031
ERX4795183	PRJEB41940	Illumina HiSeq 2500	2238212	282014712
ERX4795193	PRJEB41940	Illumina HiSeq 2500	2307456	290739456
ERX4795194	PRJEB41940	Illumina HiSeq 2500	2113368	266284368
ERX4795199	PRJEB41940	Illumina HiSeq 2500	1939050	244320300
ERX4795207	PRJEB41940	Illumina HiSeq 2500	8629598	1087329348
ERX4795209	PRJEB41940	Illumina HiSeq 2500	3108568	391679568
ERX4795211	PRJEB41940	Illumina HiSeq 2500	5020792	628508062
ERX4795215	PRJEB41940	Illumina HiSeq 2500	5628694	700264961
ERX4795220	PRJEB41940	Illumina HiSeq 2500	3413696	413756410
ERX4795224	PRJEB41940	Illumina HiSeq 2500	3888392	475325047
ERX4795230	PRJEB41940	Illumina HiSeq 2500	5057104	612910145
ERX4795236	PRJEB41940	Illumina NovaSeq 6000	6360942	942462824
ERX4795237	PRJEB41940	Illumina NovaSeq 6000	2772046	403154439
ERX4795241	PRJEB41940	Illumina NovaSeq 6000	5360694	791551703

ERX4795242	PRJEB41940	Illumina NovaSeq 6000	6333506	912768542
ERX4795246	PRJEB41940	Illumina NovaSeq 6000	5146516	723261281
ERX4795247	PRJEB41940	Illumina NovaSeq 6000	3777640	559173763
ERX4795248	PRJEB41940	Illumina NovaSeq 6000	3258280	483237014
ERX4795251	PRJEB41940	Illumina NovaSeq 6000	3514688	510147163
ERX4795256	PRJEB41940	Illumina NovaSeq 6000	11888240	1687504378
ERX4795258	PRJEB41940	Illumina NovaSeq 6000	3577648	516616767
ERX4795259	PRJEB41940	Illumina NovaSeq 6000	8679874	1281645141
ERX4795262	PRJEB41940	Illumina NovaSeq 6000	7111056	1027319277
ERX4795269	PRJEB41940	Illumina NovaSeq 6000	4002618	554209006
ERX4795275	PRJEB41940	Illumina NovaSeq 6000	9354424	1339167778
ERX4795277	PRJEB41940	Illumina NovaSeq 6000	4371166	621986240
ERX4996974	PRJEB42440	Illumina NovaSeq 6000	514302	77659602
ERX4996990	PRJEB42440	Illumina NovaSeq 6000	1175318	177473018
ERX4996993	PRJEB42440	Illumina NovaSeq 6000	1182236	178517636
ERX4997018	PRJEB42440	Illumina NovaSeq 6000	2098066	316807966
ERX5019795	PRJEB42440	Illumina NovaSeq 6000	1660258	250698958
ERX5019855	PRJEB42440	Illumina NovaSeq 6000	1632520	246510520
ERX5019860	PRJEB42440	Illumina NovaSeq 6000	1718878	259550578
ERX5019863	PRJEB42440	Illumina NovaSeq 6000	1773484	267796084
SRX1737028	PRJNA288601	Illumina MiSeq	1810875	817923354
SRX7078399	PRJNA288601	Illumina MiSeq	880192	414358092
SRX7078394	PRJNA288601	Illumina MiSeq	922257	432110955
SRX7078387	PRJNA288601	Illumina MiSeq	1346448	623956982
SRX3651390	PRJNA430813	Illumina MiSeq	2150899	1290539400
SRX2902187	PRJNA288601	Illumina MiSeq	866264	403389707
SRX7078391	PRJNA288601	Illumina MiSeq	1125876	530518704
SRX3651393	PRJNA430813	Illumina MiSeq	1581739	949043400
SRX2902042	PRJNA288601	Illumina MiSeq	799438	353606236
SRX9367473	PRJNA288601	Illumina MiSeq	763674	356478637
SRX2026408	PRJNA288601	Illumina MiSeq	1650692	777705747
SRX3651389	PRJNA430813	Illumina MiSeq	971784	583070400
SRX9367528	PRJNA288601	Illumina MiSeq	1006811	466027721
SRX2026407	PRJNA288601	Illumina MiSeq	1232400	593661087
SRX7078499	PRJNA288601	Illumina MiSeq	844991	390071440
SRX7078502	PRJNA288601	Illumina MiSeq	872088	400076210
SRX3651435	PRJNA430813	Illumina MiSeq	2447235	1468341000
SRX2902186	PRJNA288601	Illumina MiSeq	917030	423436319
SRX2026406	PRJNA288601	Illumina MiSeq	1298577	614219407
SRX7078386	PRJNA288601	Illumina MiSeq	1298751	601064806

SRX2902180	PRJNA288601	Illumina MiSeq	2733698	1261099850
SRX2902120	PRJNA288601	Illumina MiSeq	725942	333968005
SRX3651434	PRJNA430813	Illumina MiSeq	3999041	2399424600
SRX3651387	PRJNA430813	Illumina MiSeq	2378293	1426975800
SRX9367631	PRJNA288601	Illumina MiSeq	736567	339259034
SRX2026404	PRJNA288601	Illumina MiSeq	841323	398852601
SRX3651392	PRJNA430813	Illumina MiSeq	1638732	983239200
SRX3651391	PRJNA430813	Illumina MiSeq	1818450	1091070000
SRX9367629	PRJNA288601	Illumina MiSeq	559220	259047972
SRX1409801	PRJNA244549	Illumina HiSeq 2000	23197746	2319774600
SRX9367635	PRJNA288601	Illumina MiSeq	954967	436465769
SRX2026405	PRJNA288601	Illumina MiSeq	823017	391697269
SRX1997215	PRJNA288601	Illumina MiSeq	1502254	679360886
SRX7078452	PRJNA288601	Illumina MiSeq	918517	429570602
SRX3651394	PRJNA430813	Illumina MiSeq	1659196	995517600
SRX2026403	PRJNA288601	Illumina MiSeq	778442	376303902

Supplementary Figures

Supplementary Figure 1

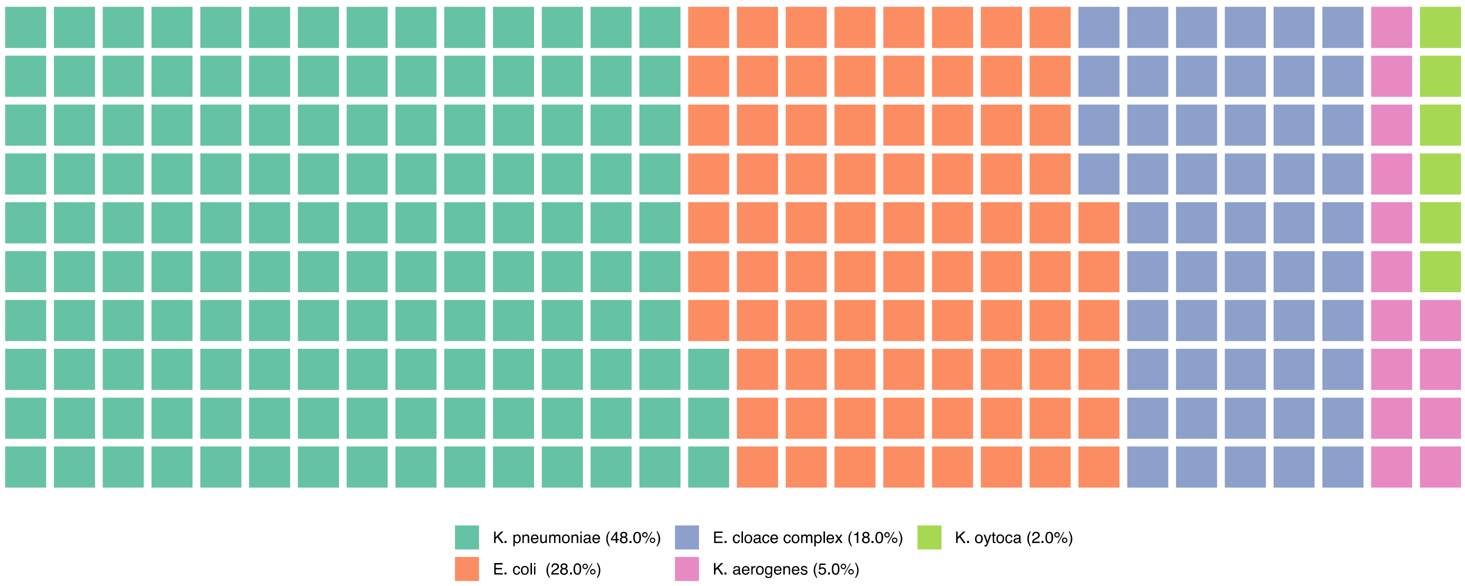

Supplementary Figure 1 legend: Waffle-plot of Carbapenem-resistant Enterobacterales cases by species collected by Georgia Emerging Infections Program between 2012-2017.

Supplementary Figure 2

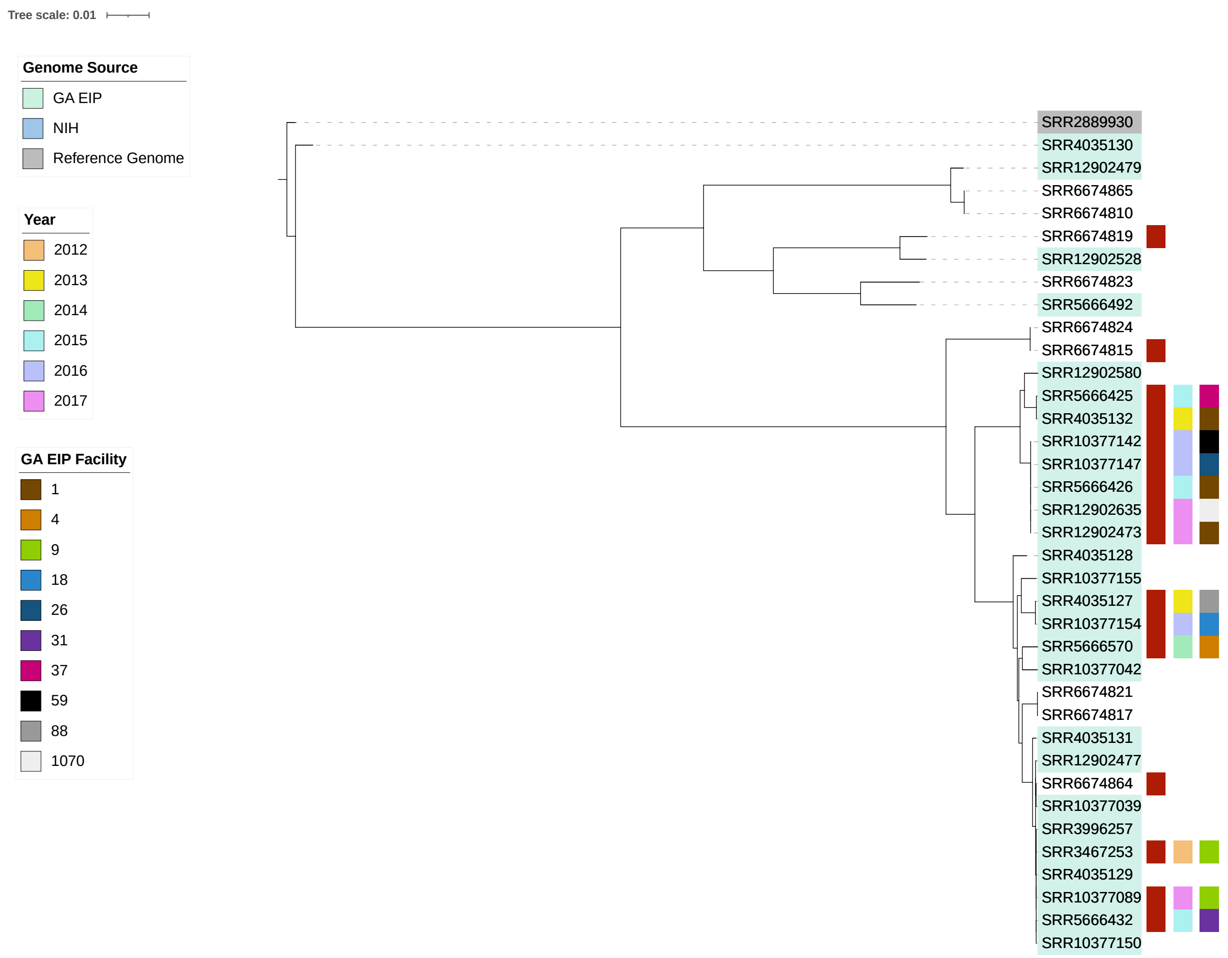

Supplementary Figure 2 legend: Phylogeny of *mcr-9* positive (n=13) and *mcr-9* negative (n=14) *E. cloacae* complex from Georgia Emerging Infections program (GA EIP) in addition to 9 available *E. cloacae* complex (three *mcr-9* positive, six *mcr-9* negative) from the National Institutes of Health. A phylogenetic tree based on a core gene alignment containing 1,904 genes defined using Roary v3.13.0. was generated using IQtree v2.0.3. A maximum likelihood tree was generated by running 1,000 bootstrap replicates under the generalized time-reversible model of evolution. The tree

was visualized and Facility IDs and year of isolation were annotated using Interactive Tree of Life (iTOL) v4. for GA EIP *mcr-9* positive genomes only.

Abbreviations: GA EIP: Georgia Emerging Infections Program, NIH: National Institutes of Health

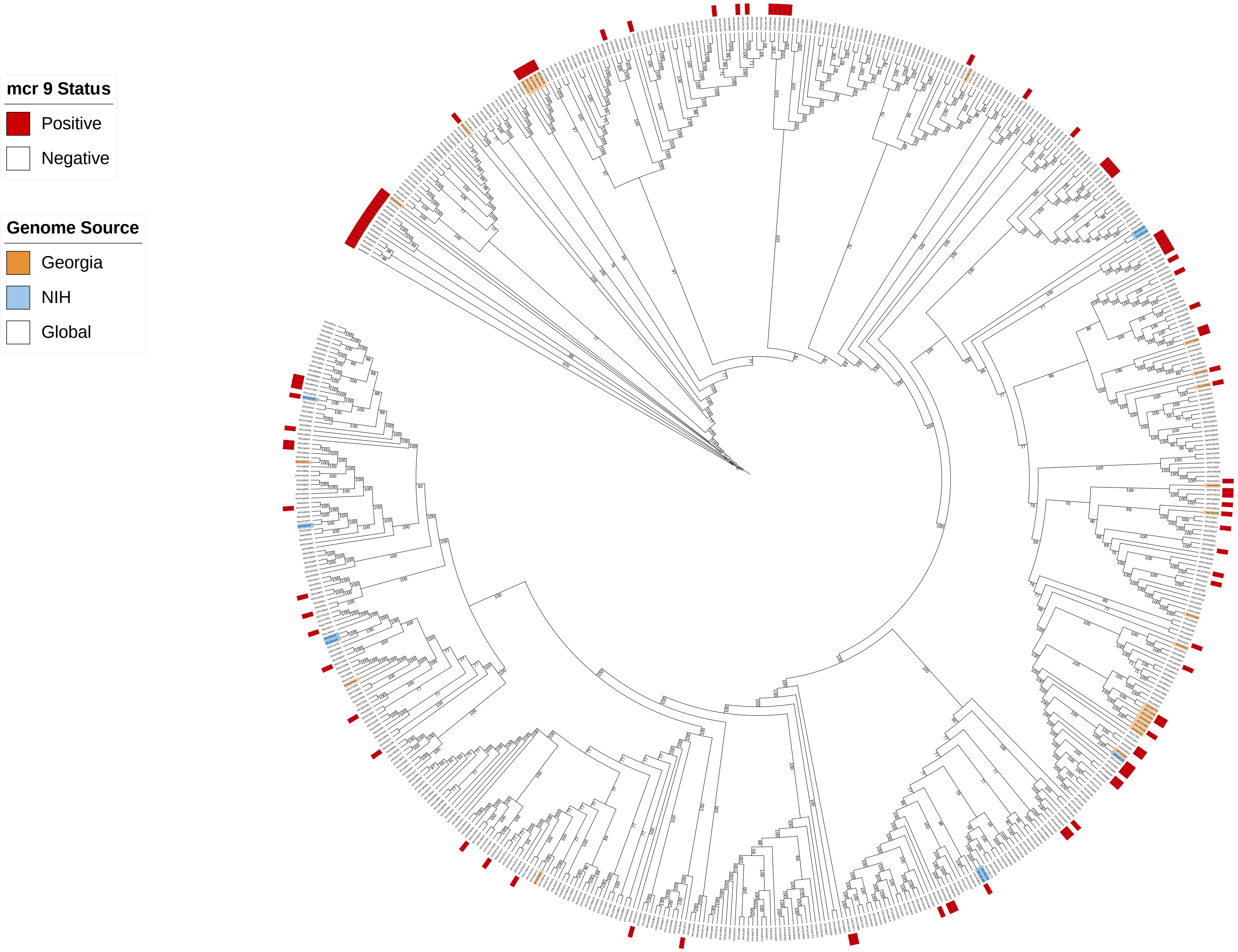

Supplementary Figure 3

Supplementary Figure 3 legend: Phylogeny of *mcr-9* positive (n=98) and *mcr-9* negative (n=510) publicly-available and Georgia Emerging Infections program (GA EIP) *E. cloacae* complex genomes. A phylogenetic tree based on a core gene alignment defined using PIRATE was generated using IQtree v2.0.3. A maximum likelihood tree was generated by running 1,000 bootstrap replicates under the generalized time-reversible model of evolution. The tree was visualized and annotated using Interactive Tree of Life (iTOL) v4. Branch lengths not to scale to allow for better visualization of clustering due to size of tree.

Abbreviations: GA EIP: Georgia Emerging Infections Program, NIH: National Institutes of Health

Supplementary Figure 4

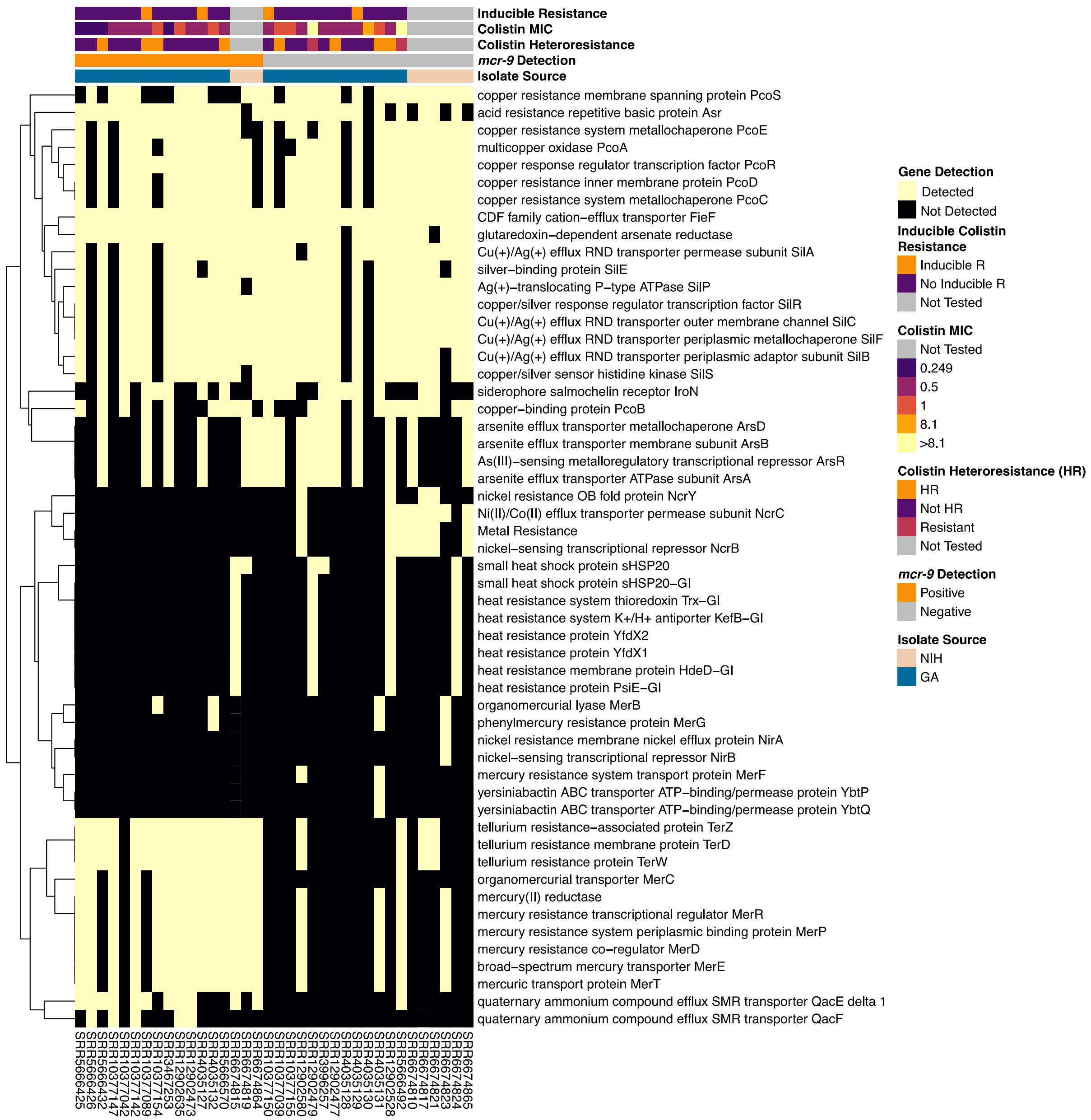

Supplementary Figure 4 Legend: Genomes were annotated using Prodigal and virulence gene content was assessed using AMRFinder. Virulence gene presence/absence heatmaps were created using the package pheatmap on R version 4.0.2 (Vienna, Austria) and the RStudio interface version 1.3.1073 (Boston, MA, USA).
